# Integrating single-cell and bulk transcriptomic analyses to develop a cancer-associated fibroblast-derived biomarker for predicting prognosis and therapeutic response in breast cancer

**DOI:** 10.3389/fimmu.2023.1307588

**Published:** 2024-01-03

**Authors:** Chunzhen Li, Lanjie Yang, Yunyan Zhang, Qianshan Hou, Siyi Wang, Shaoteng Lu, Yijie Tao, Wei Hu, Liyuan Zhao

**Affiliations:** ^1^ National Key Laboratory of Immunity & Inflammation, Naval Medical University, Shanghai, China; ^2^ Department of Breast Surgery, Changhai Hospital, Naval Medical University, Shanghai, China; ^3^ Department of Respiratory and Critical Care Medicine, Changzheng Hospital, Naval Medical University, Shanghai, China; ^4^ Department of Anesthesia Physiology, Naval Medical University, Shanghai, China

**Keywords:** breast cancer, cancer-associated fibroblast, prognostic signature, ScRNA-seq, tumor microenvironment

## Abstract

**Background:**

Cancer-associated fibroblasts (CAFs) contribute to the progression and treatment of breast cancer (BRCA); however, risk signatures and molecular targets based on CAFs are limited. This study aims to identify novel CAF-related biomarkers to develop a risk signature for predicting the prognosis and therapeutic response of patients with BRCA.

**Methods:**

CAF-related genes (CAFRGs) and a risk signature based on these genes were comprehensively analyzed using publicly available bulk and single-cell transcriptomic datasets. Modular genes identified from bulk sequencing data were intersected with CAF marker genes identified from single-cell analysis to obtain reliable CAFRGs. Signature CAFRGs were screened via Cox regression and least absolute shrinkage and selection operator (LASSO) analyses. Multiple patient cohorts were used to validate the prognosis and therapeutic responsiveness of high-risk patients stratified based on the CAFRG-based signature. In addition, the relationship between the CAFRG-based signature and clinicopathological factors, tumor immune landscape, functional pathways, chemotherapy sensitivity and immunotherapy sensitivity was examined. External datasets were used and sample experiments were performed to examine the expression pattern of MFAP4, a key CAFRG, in BRCA.

**Results:**

Integrated analyses of single-cell and bulk transcriptomic data as well as prognostic screening revealed a total of 43 prognostic CAFRGs; of which, 14 genes (TLN2, SGCE, SDC1, SAV1, RUNX1, PDLIM4, OSMR, NT5E, MFAP4, IGFBP6, CTSO, COL12A1, CCDC8 and C1S) were identified as signature CAFRGs. The CAFRG-based risk signature exhibited favorable efficiency and accuracy in predicting survival outcomes and clinicopathological progression in multiple BRCA cohorts. Functional enrichment analysis suggested the involvement of the immune system, and the immune infiltration landscape significantly differed between the risk groups. Patients with high CAF-related risk scores (CAFRSs) exhibited tumor immunosuppression, enhanced cancer hallmarks and hyposensitivity to chemotherapy and immunotherapy. Five compounds were identified as promising therapeutic agents for high-CAFRS BRCA. External datasets and sample experiments validated the downregulation of MFAP4 and its strong correlation with CAFs in BRCA.

**Conclusions:**

A novel CAF-derived gene signature with favorable predictive performance was developed in this study. This signature may be used to assess prognosis and guide individualized treatment for patients with BRCA.

## 1 Introduction

Breast cancer (BRCA) is one of the most common cancers globally, with 2.3 million new cases being reported in 2020, and the leading cause of cancer-related death among women (1). Advancements in diagnostic approaches and surgery-based comprehensive treatments (such as neoadjuvant chemoradiotherapy, hormonal therapy, and targeted therapy) have reduced the mortality rate of BRCA. However, some patients still have a poor prognosis owing to tumor metastasis, recurrence and poor therapeutic responsiveness (2). With more attention being paid to immunotherapies, novel therapeutic approaches involving immune checkpoint blockade (ICB) therapy, adoptive cell therapy (ACT), immunomodulatory agents and tumor vaccines have been developed for the treatment of several complex and advanced cancers such as BRCA, lung cancer and melanoma (3–5). Although immune checkpoint inhibitors such as pembrolizumab and atezolizumab have some therapeutic benefits in clinical practice, the proportion of responsive patients remains low. In addition, reliable tools for predicting the treatment response of patients are limited, necessitating the identification of novel therapeutic targets for BRCA and novel biomarkers that can be used to assess the prognosis and treatment response of patients with BRCA (6–8).

The tumor microenvironment (TME) is mainly composed of infiltrative immune cells, stromal cells and secretory signaling factors that are closely associated with the biological behavior of tumors (9, 10). Numerous recent studies have demonstrated that TME components such as cancer-associated fibroblasts (CAFs) and macrophages can limit the intra-tumoral infiltration of effector immune cells directly by forming a spatial barrier-like structure, leading to immunotherapeutic resistance (11). Furthermore, these cells can interact with tumor cells and induce a suppressive microenvironment, thereby promoting cancer progression indirectly (12, 13). Therefore, investigating the components of TME and their interactions with BRCA cells is important for elucidating tumor–immune regulatory mechanisms and identifying promising therapeutic targets.

CAFs are the predominant members of the stromal population in TME. They could regulate various biological processes such as extracellular matrix remodeling, secretory signaling, and the crosstalk between multiple cell types (10, 13, 14). Studies have shown that CAFs could promote the malignant characteristics of BRCA, including proliferation, angiogenesis, metastasis and treatment resistance, further resulting in an unfavorable prognosis (14–16). For example, CAF-derived secreted factors such as transforming growth factor-β (TGF-β), hepatocyte growth factor (HGF), fibroblast growth factor 5 (FGF5), and even interleukins (ILs) and chemokines, could facilitate BRCA progression and treatment resistance through different mechanisms (15). In addition, CAFs could also influence the spatial distribution and limit the function of effector immune cells, to indirectly promote survival of tumor cells (15). A recent study based on pan-cancer bulk and single-cell transcriptomic analyses identified the CAF-derived secreted protein BGN as a poor prognostic factor for BRCA, and the association between its high expression and immunotherapy resistance was validated in samples from a real-world ICB cohort (17). Consequently, targeting CAFs and CAF-related genes has emerged as a promising therapeutic strategy for BRCA.

Although some studies have focused on the function of CAFs in BRCA, studies addressing the prognostic value of CAF-related genes and their relationship with TME and therapeutic response in BRCA are limited. The emergence of single-cell RNA sequencing (scRNA-seq) has dramatically improved the precision of studies on TME, enabling the acquisition of deeper cellular and molecular information (18). Therefore, we speculated that integrating single-cell and bulk transcriptomic analyses might help to develop a reliable CAF-based risk signature for predicting the prognosis and therapeutic response of patients with BRCA.

**Figure 1:**
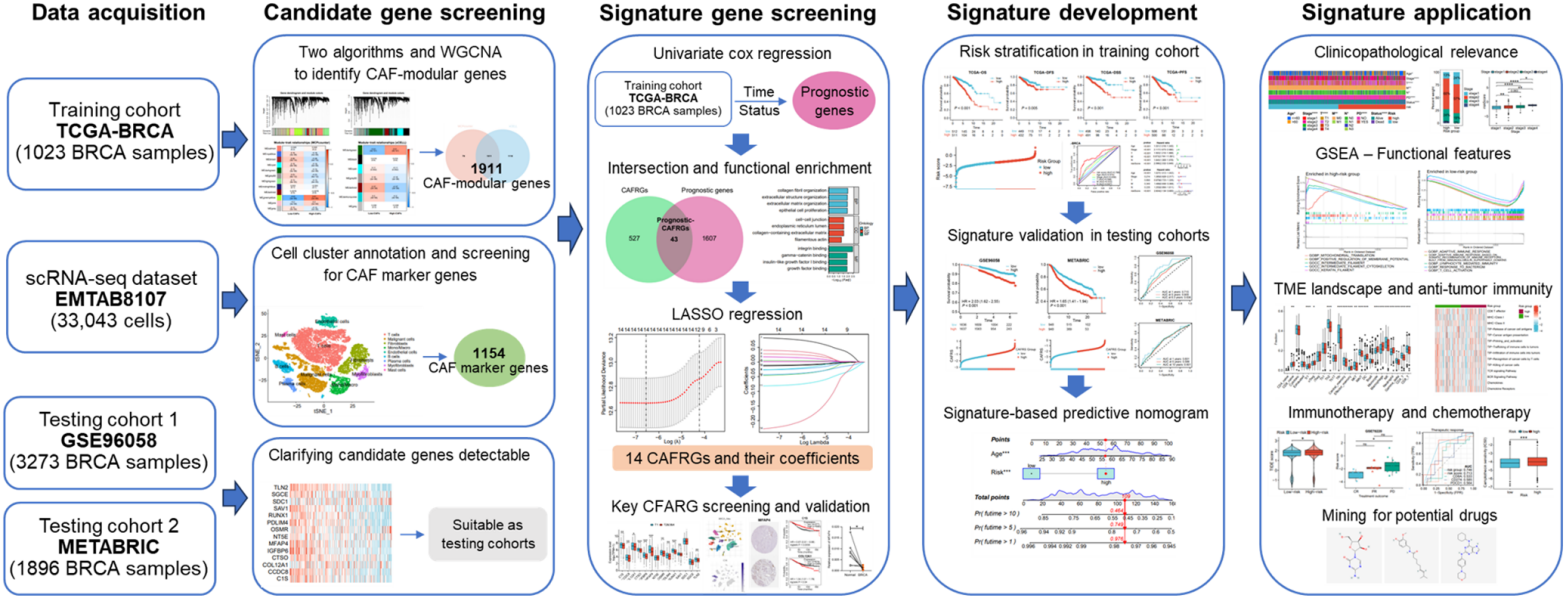
Flowchart demonstrating the protocol of this study.

In this study, we comprehensively analyzed scRNA-seq and bulk transcriptomic data to screen for CAF-related genes (CAFRGs) associated with the prognosis of BRCA and developed a novel risk signature based on these CAFRGs. The predictive performance and clinicopathological relevance of the risk signature were validated in multiple patient cohorts. In addition, the utility of the signature in profiling the TME landscape, immune function and therapeutic susceptibility was assessed. The overall protocol of this study is illustrated in **Figure 1**.

## 2 Materials and methods

### 2.1 Data sources and processing

The bulk RNA-seq and clinical data of 1109 BRCA samples and 113 normal samples were extracted from The Cancer Genome Atlas (TCGA) database (https://portal.gdc.cancer.gov/) (TCGA-BRCA cohort). After duplicates and cases with an overall survival of<60 days were removed, 1023 BRCA samples were subjected to subsequent analysis. A quality-controlled scRNA-seq dataset of primary BRCA tissues based on the 10x Genomics platform, EMTAB8107 (containing data on 33,043 cells from 14 untreated patients), was obtained from Tumor Immune Single-cell Hub 2 (TISCH2, http://tisch.comp-genomics.org/home/) (19, 20). The “Seurat” package was used for data analysis, and after data normalization (log normalization with a scale factor of 10000), 2000 highly variable genes were selected for principal component analysis (PCA) using the “FindVariableFeatures” and “runPCA” functions (21). Cell clusters were identified based on the first 20 principal components (PCs) using the t-SNE algorithm, with the resolution set to 0.5. Differentially expressed genes between the cell clusters were identified using the ‘FindAllMarkers’ function, and cell populations were annotated using the “SingleR” algorithm and reference-based annotation in the CellMarker2.0 database. Two independent BRCA datasets, namely, GSE96058 (N = 3273) and METABRIC (N = 1896), were used for external validation of the risk signature, and their clinical information was integrated to assess the clinicopathological relevance of the risk signature.

### 2.2 Assessment of CAF abundance and survival analysis

Data on the abundance of tumor-infiltrating immune cells in TCGA-BRCA samples were extracted from the “immune estimation” module of the TIMER2.0 database (http://timer.cistrome.org/). In addition, abundance data of CAFs quantified using the xCELL and MCPcounter algorithms were extracted for subsequent grouping (22). The ‘survminer’ and ‘survival’ R packages were used to evaluate the optimal cutoff value of the abundance of CAFs for survival analysis. Subsequently, Kaplan–Meier curves were plotted to demonstrate differences in survival between CAF groups.

### 2.3 Identification of CAF-related genes with prognostic value

CAF-related genes (CAFRGs) were identified through single-cell and bulk transcriptomic analyses. After cell annotation, the marker genes of CAF populations were retained. Weighted gene co-expression network analysis (WGCNA) was performed to screen for modules and genes most relevant to CAFs. After samples were clustered and outliers were removed, a soft-thresholding value was determined to achieve optimal operational efficiency. Genes were divided into different modules, with the number of genes in each module being >50. Subsequently, correlation analysis was performed to screen for modules that were most relevant to CAFs. The genes obtained from the most relevant modules were intersected with the marker genes of CAFs obtained from scRNA-seq data to screen for reliable CAFRGs. Univariate Cox analysis was performed to identify CAFRGs with prognostic value (*P*< 0.05) using the “survival” package.

### 2.4 Development and validation of a CAF-related risk signature

Prognostic CAFRGs were subjected to multivariate Cox and LASSO regression analyses in the TCGA-BRCA cohort to determine the ideal CAFRGs and their coefficients for establishing a risk signature. Each sample was assigned a CAF-related risk score (CAFRS) using the following formula:


$$\text{CAFRS}=\sum_{i=1}^{n}\left[\text{coefficient }(\text{CAFRGi})\;*\text{ expression }(\text{CAFRGi})\right]$$

Patients in each cohort were stratified into a high-CAFRS group and a low-CAFRS group based on the median CAFRS as previously described (23). The “pheatmap” package was used to visualize the distribution of risk scores and the expression profiles of signature CAFRGs. Survival differences between the two CAFRS groups were demonstrated utilizing the same packages as mentioned above. Additionally, signature CAFRGs were also subjected to consensus clustering analysis.

### 2.5 Clinicopathological relevance analysis and establishment of a nomogram

Multi-index and time-dependent receiver operating characteristic (ROC) curves were plotted to evaluate the accuracy of CAFRSs in predicting the survival of patients. Multiple clinicopathological parameters were compared between the two CAFRS groups to examine the relationship between CAFRSs and the clinicopathological parameters. Univariate and multivariate Cox regression analyses were performed to assess whether CAFRSs and the clinicopathological parameters served as independent indicators of prognosis in BRCA cohorts. Subsequently, a nomogram integrating CAFRSs and clinicopathological parameters was developed using the “nomogram” function to predict risk and survival quantitatively. The “rms”, “timeROC”, “ComplexHeatmap” and “ggplot2” packages were used for the abovementioned analyses.

### 2.6 Characterization of functional phenotypes

Gene set enrichment analysis (GSEA) was performed to screen for biological processes and pathways associated with CAFRGs in BRCA. For Gene Ontology (GO) and Kyoto Encyclopedia of Genes and Genomes (KEGG) enrichment analyses, gene sets were obtained from the MSigDB. GSEA was performed using the “org.Hs.eg.db” and “clusterProfiler” packages (24). The screening criteria for significantly enriched items included a |normalized enrichment score (NES)| > 1, a p-value< 0.05, and a false discovery rate (FDR)< 0.25.

### 2.7 Assessment of the tumor immune landscape

To assess the immune characteristics of the two CAFRS groups, the abundance of tumor-infiltrating immune cells (TIICs) was evaluated, and microenvironmental scores, tumor stemness scores and tumor immunity-related signature scores were calculated. The CIBERSORT and ImmunecellAI algorithms were used to quantify the intra-tumoral abundance of immune cells (25, 26). The CIBERSORT analysis was performed according to the officially recommended parameters and repeated 1000 times. Results of ImmunecellAI were obtained using the function of immune cell abundance analysis on the ImmunecellAI platform (http://bioinfo.life.hust.edu.cn/ImmuCellAI#!/), following the official tutorials. The correlation between the expression of signature CAFRGs and the infiltration levels of immune cells was assessed using the Spearman method. Additionally, the ESTIMATE algorithm was used to evaluate immune scores, stromal scores, ESTIMATE scores and tumor purity. The immunological profiles of patients in the two CAFRS groups were assessed using the GSVA algorithm and the scoring system based on the IOBR package (27). Differences between the two CAFRS groups were visualized on heat maps and box plots.

### 2.8 Therapeutic response analysis and drug screening

Based on the results of functional enrichment and immune infiltration analyses, immune regulatory molecules (such as antigen-presenting molecules and immune checkpoint molecules) and immunotherapy response scoring systems (Tumor Immune Dysfunction and Exclusion [TIDE] scores and immunophenoscores [IPSs]) were integrated to examine the relationship between CAFRS and immunotherapy response (28, 29). In addition, transcriptomic and survival analyses were performed to validate the results in a real-world patient cohort undergoing anti-PD-1 ICB therapy (GSE78220) (30).

The “pRRophetic” package was used to evaluate the half-maximal inhibitory concentration (IC50) of common clinical chemotherapeutic or targeted drugs, such as cisplatin, docetaxel and axitinib, to compare drug sensitivity between the high- and low-CAFRS groups (31). Transcriptomic differences between the two CAFRS groups were assessed and submitted to the Connectivity Map (Cmap, https://clue.io/) platform to identify promising drugs for treating high-CAFRS BRCA (32).

### 2.9 Screening and validation of key CAFRGs

To screen for CAFRGs with biological significance in BRCA, the expression of signature CAFRGs was compared between BRCA and normal tissue samples, and key CAFRGs were further identified via clinicopathological and proteomic analyses (33). Two independent scRNA-seq datasets (GSE176078 and GSE114727) from the TISCH2 database were used to verify the expression of key CAFRGs in different cell populations (19). To validate the expression of key CAFRGs at the protein level, publicly available immunohistochemical (IHC) images and proteomic data were obtained from the Human Protein Atlas (HPA) and Clinical Proteomic Tumor Analysis Consortium (CPTAC) databases, respectively (34). The KM Plotter was used to examine the role of key CAFRGs in prognosis in external BRCA cohorts (35). The Gene Expression Profiling Interactive Analysis (GEPIA) database and a transcriptomic dataset (GSE22820) including 176 primary BRCA tissue samples and 10 normal breast tissue samples were used to examine the expression of microfibrillar-associated protein 4 (MFAP4), a key CAFRG (36, 37). For dataset GSE22820, differential expression analysis was performed following the descriptions of previous studies, with thresholds for differences defined as |log_2_FC| > 1 and the p-value< 0.01 (38).

### 2.10 *In vitro* assays for assessing the expression and tissue distribution of MFAP4

Real-time quantitative reverse transcription polymerase chain reaction (qRT-PCR) and IHC analysis were performed to evaluate the expression of MFAP4 in BRCA and adjacent normal tissues. Briefly, seven pairs of fresh BRCA tissue samples and corresponding paracancerous tissue samples were obtained from the Department of Thyroid and Breast Surgery, the First Affiliated Hospital of Naval Medical University. Informed consent was obtained from all patients. The fresh tissues were rapidly frozen and stored in liquid nitrogen until RNA extraction. Total RNA was extracted from tissues using the TRIzol reagent according to the manufacturer’s instructions. The reverse transcription and real-time PCR experiments were performed as described previously (39). The mRNA expression of MFAP4 was evaluated and normalized to that of GAPDH. The primers used for PCR were synthesized by Sangon Biotech (Shanghai, China), and the sequences are listed in **Supplementary Table 1**. IHC staining was performed using primary antibodies against MFAP4 (GB113768-100) and alpha-smooth muscle actin (α-SMA) (GB111364-100) (Servicebio, Wuhan, China) as described previously (39).

### 2.11 Statistical analysis

The R software (version 4.1.2) and several online tools such as TISCH, TIMER2.0 and Cmap, were used for statistical analysis. The “limma”, “ggplot2”, “Seurat”, “WGCNA”, “survival”, “pheatmap”, “GSVA” and “pRRophetic” packages were used in this study, and their application situations have been described in respective sections. The Student’s t-test and chi-square test were used to compare continuous and categorical variables, respectively. The Wilcoxon test was used to compare gene expression between groups. In addition, Spearman analysis was performed to assess the correlation between different variables. A p-value of< 0.05 was considered statistically significant.

## 3 Results

### 3.1 Dual WGCNA for the identification of CAFRGs in BRCA

**Figure 2:**
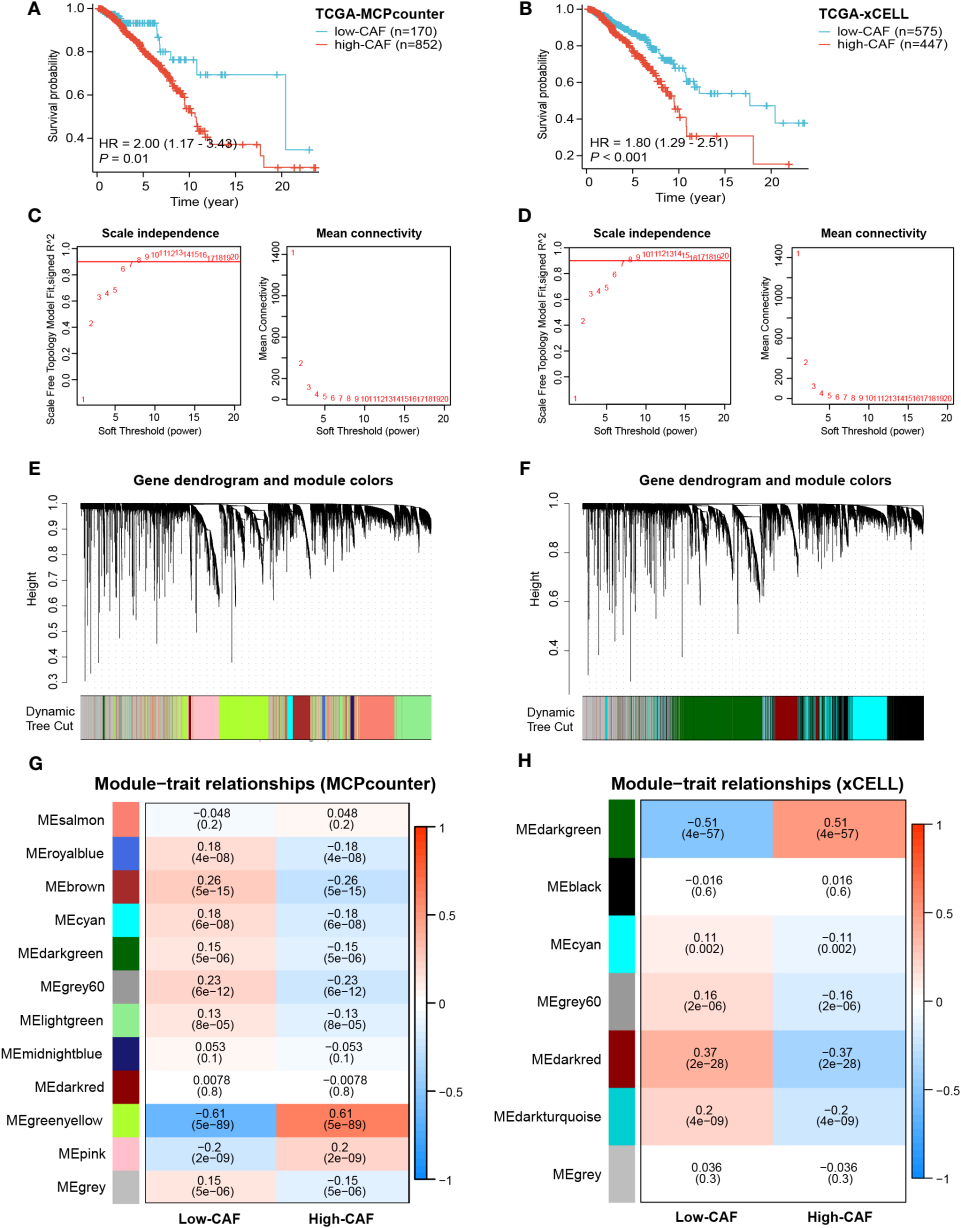
Prognostic value of CAFs and screening of modular genes most relevant to CAF infiltration using two algorithms. **(A, B)** Survival analysis showed significant differences in prognosis between the high- and low-CAF-infiltration groups. **(C, D)** The optimal soft-thresholding power was selected as 7 in WGCNA. **(E–H)** Hierarchical clustering was performed to distinguish different modules and identify modules most relevant to CAFs. The results displayed on the left panel **(A, C, E, G)** are based on the MCPcounter algorithm, and those displayed on the right panel **(B, D, F, H)** are based on the xCELL algorithm.

The MCPcounter and xCELL algorithms were used to quantify tumor-infiltrating immune cells (TIICs) in TCGA-BRCA cohort. Patients were divided into high- and low-CAF-infiltration groups for survival analysis. As anticipated, patients with a high abundance of tumor-infiltrating CAFs had significantly shorter survival (*P<* 0.01 and *P<* 0.001, **Figures 2A, B**). These results suggest that CAFs play a role in the prognosis of BRCA. Furthermore, dual WGCNA was performed to identify modules and genes most closely associated with CAFs in BRCA. Based on the optimal soft-thresholding power of 7 (**Figures 2C, D**), several gene modules were identified (**Figures 2E, F**). The green-yellow and dark-green modules were found to be most closely associated with the abundance of CAFs calculated by MCPcounter and xCELL, respectively (correlation = 0.61, *P*< 0.001; correlation = 0.51, *P*< 0.001; **Figures 2G, H**; **Supplementary Figure 1**). A total of 1990 genes in the green-yellow module and 3029 genes in the dark-green module were extracted for subsequent analyses.

### 3.2 ScRNA-seq data analysis to identify marker genes of CAFs

**Figure 3:**
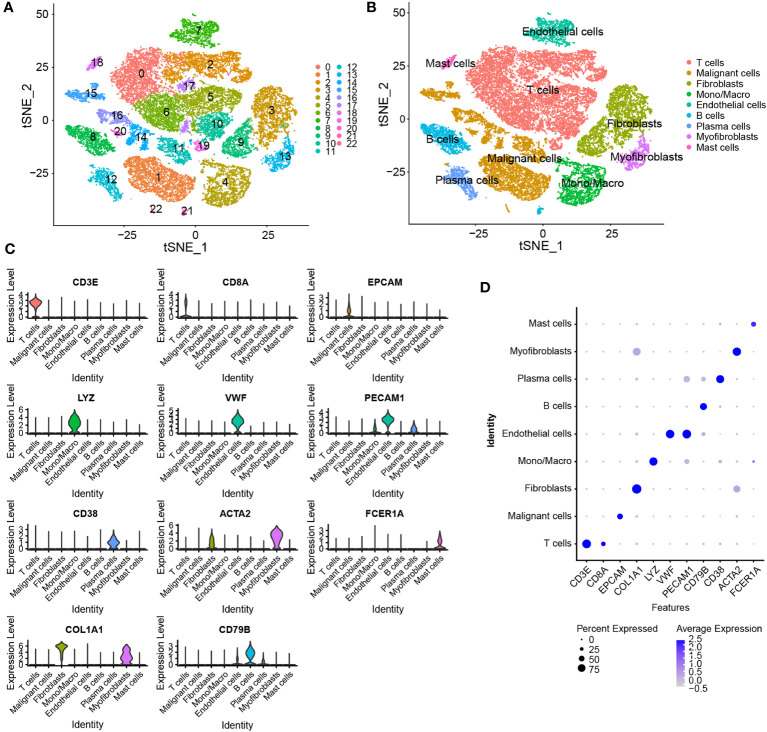
Identification of marker genes of CAFs using scRNA-seq data. **(A)** t-SNE plot of 23 cell clusters grouped using the Seurat package. **(B)** The t-SNE plot of annotated cell types. **(C, D)** Violin and bubble plots demonstrating the differential expression of marker genes in different cell types.

The Seurat package was used to normalize the scRNA-seq data. As shown in the t-SNE map in **Figure 3A**, a total of 33,043 cells were grouped into 23 clusters based on the top 2000 highly variable genes and the top 20 PCs. Based on common cell markers, the 23 clusters were annotated into nine cell types, including T cells, malignant cells, fibroblasts, monocytes/macrophages, endothelial cells, B cells, plasma cells, myofibroblasts and mast cells, using the SingleR algorithm (**Figure 3B**). Clusters 3 and 9 were identified as fibroblasts, that is, CAFs. The expression of marker genes in different cell types was visualized on a violin plot, bubble plot, t-SNE map and heat map (**Figures 3C, D**; **Supplementary Figures 2A, B**). Eventually, 1154 marker genes of CAFs were selected by comparing gene expression among the nine cell types.

### 3.3 Screening for prognostic CAFRGs and development of a CAFRG-based risk signature

**Figure 4:**
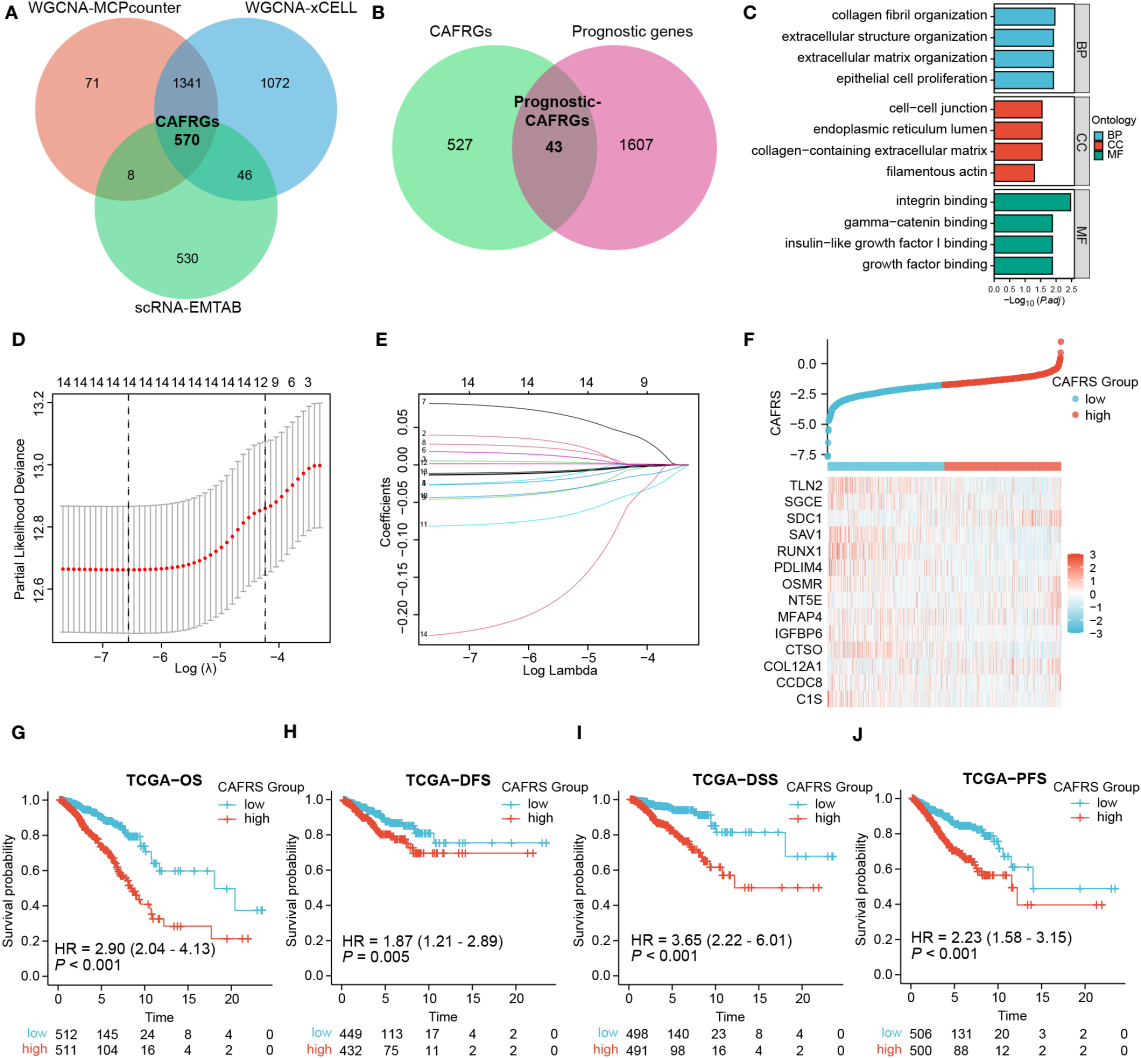
Screening of CAF-related genes (CAFRGs) and establishing a CAF-related risk signature in TCGA-BRCA cohort. **(A)** CAFRGs were identified via WGCNA and scRNA-seq data analyses. **(B)** Prognostic CAFRGs were identified by intersecting CAFRGs with prognostic genes. **(C)** Functional enrichment analysis of prognostic CAFRGs. **(D, E)** LASSO analysis was performed to determine candidate CAFRGs and their coefficients for establishing the risk signature. **(F)** Distribution of CAF-related scores (CAFRSs) and expression of signature CAFRGs in two groups. **(G–J)** Analysis of different survival types showed that patients with high CAFRSs had a worse prognosis.

The 1990 genes in the green-yellow module, 3029 genes in the dark-green module and 1154 marker genes of CAFs were intersected to obtain 570 CAFRGs (**Figure 4A**). Univariate Cox regression analysis revealed 1650 genes associated with the prognosis of BRCA. These genes were intersected with the 570 CAFRGs to obtain 43 prognostic CAFRGs (**Figure 4B**; **Supplementary Table 2**). These prognostic CAFRGs were primarily enriched in cellular components such as collagen and extracellular matrix and related biological processes as well as molecular functions such as integrin binding and growth factor binding (**Figure 4C**). Based on the results of LASSO regression analysis, 14 prognostic CAFRGs and their respective coefficients were used to establish a CAF-related risk signature (**Figures 4D, E**; **Table 1**).

**Table 1:** Coefficients of CAFRGs used for developing the risk signature.

Gene symbol	Description	Coefficient
C1S	Complement C1s	-0.0124
CCDC8	Coiled-coil domain-containing 8	0.0376
COL12A1	Collagen type XII alpha 1 chain	0.0048
CTSO	Cathepsin O	-0.0248
IGFBP6	Insulin-like growth factor-binding protein 6	-0.0232
MFAP4	Microfibril-associated protein 4	0.0161
NT5E	Ecto-5’-nucleotidase	0.0783
OSMR	Oncostatin M receptor	0.0262
PDLIM4	PDZ and LIM domain 4	-0.0430
RUNX1	RUNX family transcription factor 1	-0.0411
SAV1	Salvador family WW domain-containing protein 1	-0.0785
SDC1	Syndecan 1	0.0015
SGCE	Sarcoglycan epsilon	-0.0104
TLN2	Talin 2	-0.2146

The 14 CAFRGs used to construct the CAF-related risk signature included C1s, CCDC8, COL12A1, CTSO, IGFBP6, MFAP4, NT5E, OSMR, PDLIM4, RUNX1, SAV1, SDC1, SGCE and TLN2. The CAFRS of each sample in the TCGA-BRCA cohort was calculated, and patients were divided into high- and low-CAFRS groups based on the median risk score (-1.748) (**Figure 4F**). As anticipated, the heat map showed that the expression of protective CAFRGs was higher in the low-CAFRS group, whereas that of harmful CAFRGs was higher in the high-CAFRS group (**Figure 4F**). Subsequently, the CAF-related risk signature was used for survival analysis. Kaplan–Meier curves revealed that overall survival (OS), disease-free survival (DFS), disease-specific survival (DSS) and progression-free survival (PFS) were consistently longer in the low-CAFRS group than in the high-CAFRS group, suggesting an improved prognosis for low-CAFRS patients (*P*< 0.01) (**Figures 4G–J**).

### 3.4 Performance evaluation and external validation of the CAF-related risk signature

**Figure 5:**
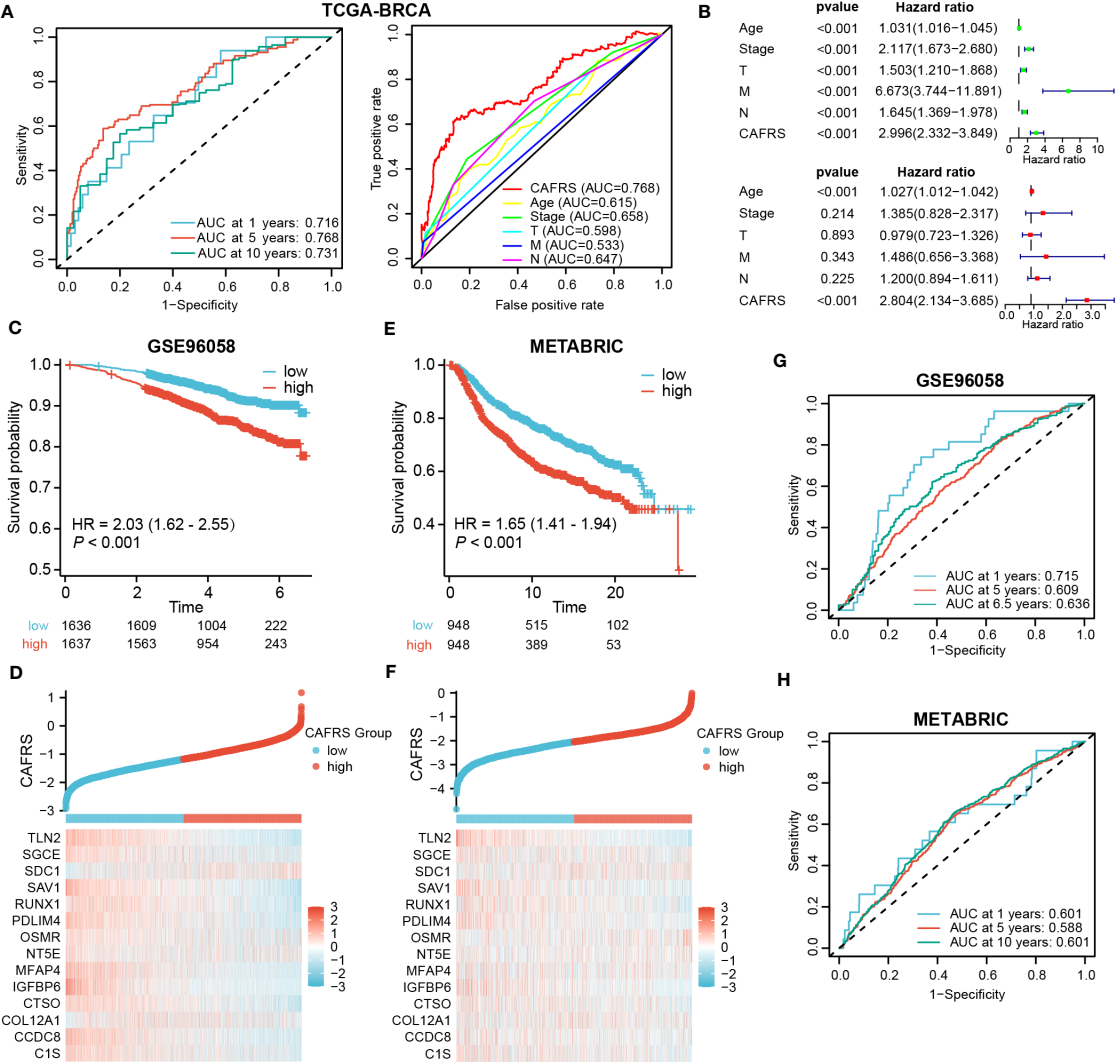
Evaluation and external validation of the CAF-related risk signature. **(A)** ROC curves demonstrating the predictive performance of the signature. **(B)** Univariate and multivariate analyses of the CAFRS in TCGA-BRCA cohort. **(C, E)** Kaplan–Meier curves demonstrating a worse prognosis of high-CAFRS patients in two external BRCA cohorts. **(D, F)** Distribution of CAF-related scores (CAFRSs) and expression profiles of signature CAFRGs in GSE96058 **(D)** and METABRIC **(F)** cohorts. **(G, H)** ROC curves demonstrating the predictive performance of the signature in two external BRCA cohorts.

Multi-index and time-dependent ROC curves were plotted to assess the predictive accuracy of the CAF-related risk signature. The area under the ROC curve (AUC) at 1, 5 and 10 years was 0.716, 0.768 and 0.731, respectively, suggesting that the predictive performance of the risk signature was superior to that of other clinical indicators (**Figure 5A**). Univariate and multivariate regression analyses revealed that CAFRS was an independent indicator of prognosis in BRCA, with the results being consistent across multiple types of survival (*P*< 0.001) (**Figure 5B**; **Supplementary Figures 3A, B**). The predictive accuracy of the risk signature was validated in two external cohorts, GSE96058 and METABRIC (**Supplementary Figures 3C, D**). The expression profiles of signature CAFRGs in these two groups were consistent with those in the training cohort. (**Figures 5D, F**). Patients in the high-CAFRS group in both cohorts had significantly shorter survival (*P*< 0.001) (**Figures 5C, E**). In addition, ROC curves demonstrated modest predictive performance of the CAF-related risk signature in the two external cohorts (**Figures 5G, H**; **Supplementary Figures 4A, B**).

Furthermore, we divided patients into different clinical subgroups according to their clinicopathologic characteristics to test whether this signature could continue to be prognostic in each independent subgroup. **Supplementary Figures 5A, B** showed that high-CAFRS patients still suffered worse OS, regardless of the clinical subgroup. Altogether, these results suggest that the CAF-related risk signature is a reliable tool for predicting the prognosis of BRCA.

### 3.5 Strong association between the risk signature and clinicopathological indicators

**Figure 6:**
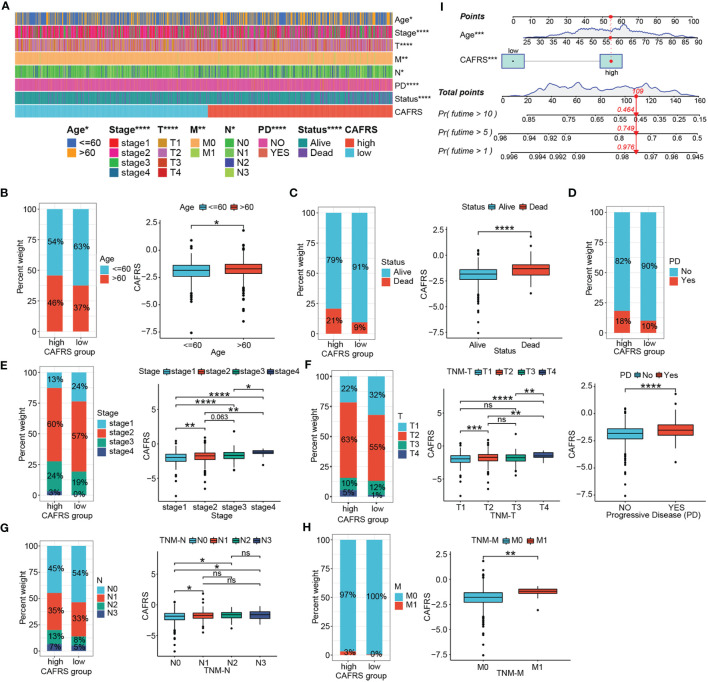
Clinicopathological relevance of the CAF-related risk signature. **(A)** Heat map of patients with different clinicopathological characteristics in the high- and low-CAFRS groups. **(B–H)** Histograms and box plots demonstrating the association between the CAFRS and clinicopathological parameters including age **(B)**, survival status **(C)**, disease progression **(D)**, tumor stage **(E)**, TNM-T stage **(F)**, TNM-N stage **(G)** and TNM-M stage **(H)**. **(I)** CAFRS-based nomogram for predicting patient prognosis quantitatively. (ns: not significant, *P < 0.05, **P < 0.01, ***P < 0.001, ****P < 0.0001).

This signature correlated well with clinicopathological indicators of BRCA patients. As shown in **Figure 6A**, indicators such as age, survival outcome and tumor TNM stage significantly differed between the high- and low-CAFRS groups. Notably, patients with advanced age, poor survival outcomes and progressive disease had significantly higher CAFRSs (**Figures 6B–D**). Correlation analysis revealed a positive relationship between CAFRSs and TNM staging indexes, indicating the favorable clinicopathological relevance of the CAFRS (**Figures 6E–H**). These results were validated in the two external cohorts, in which patients with advanced age, poorer outcomes, larger tumors, higher tumor grades and more positive lymph nodes detected (LN^+^) were found to have higher CAFRSs (**Supplementary Figures 4C–I, J, K**). Moreover, high-CAFRS patients in the METABRIC cohort also experienced significantly shorter relapse-free survival (RFS) (*P*< 0.05) (**Supplementary Figures 4L**). Subsequently, we combined the CAFRS and the independent prognostic factor (age) to establish a nomogram for quantitative prediction of patient survival (**Figure 6I**).

### 3.6 GSEA implied the association of tumor immunity with the risk signature

**Figure 7:**
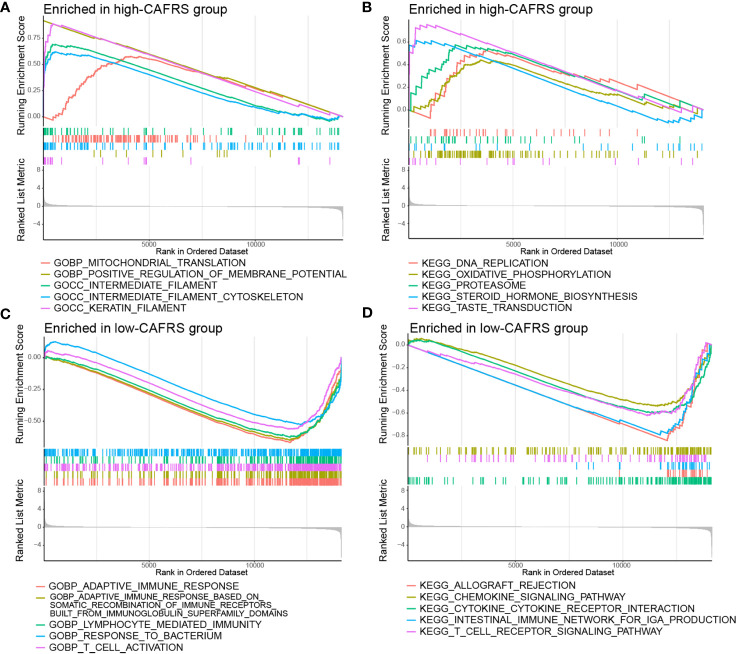
Gene set enrichment analysis. Significantly enriched GO terms and KEGG pathways in the high-CAFRS group **(A, B)** and the low-CAFRS group **(C, D)**.

GSEA was performed to investigate the underlying causes of differences in prognosis and clinical characteristics between the high- and low-CAFRS groups. High-CAFRS tumors were mainly associated with biological processes and pathways related to mitochondrial translation, DNA replication, oxidative phosphorylation and glycolysis (**Figures 7A, B**; **Supplementary Figure 6A**). Low-CAFRS tumors were closely associated with tumor immunity, including adaptive immune responses, T-cell activation, interferon-gamma responses, cytokine–receptor interaction and lymphocyte-mediated immunity (**Figures 7C, D**; **Supplementary Figure 6B**). These results suggest that antitumor immunity and the tumor immune microenvironment may contribute to the differential prognosis of patients with BRCA.

### 3.7 Deciphering the TME landscape in different CAFRS groups

**Figure 8:**
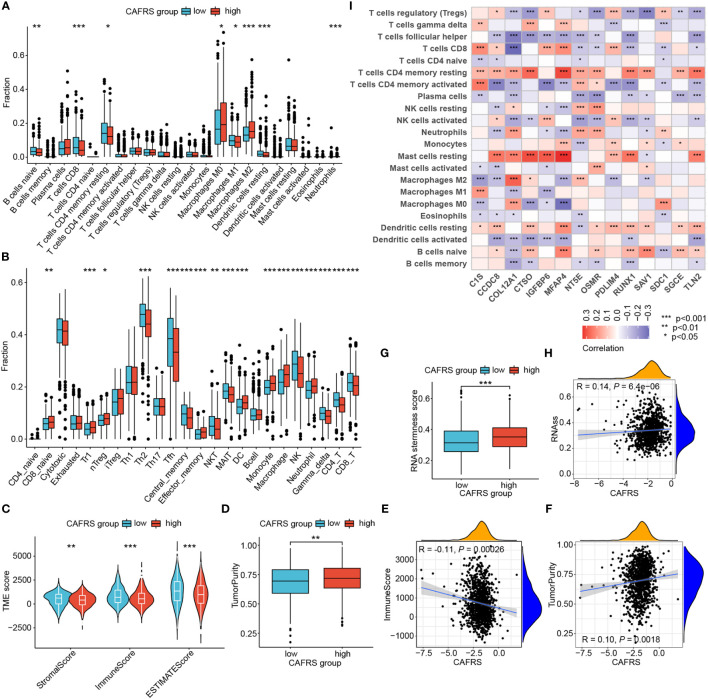
Assessment of the tumor microenvironment (TME) landscape. **(A, B)** The abundance of TIICs in the high- and low-CAFRS groups was evaluated using the CIBERSORT **(A)** and ImmunecellAI **(B)** algorithms. **(C, D)** Microenvironmental scores in the two CAFRS groups were calculated using the ESTIMATE algorithm. **(E, F)** Correlation of CAFRSs with immune scores and tumor purity. **(G)** Differences in stemness scores between the two CAFRS groups. **(H)** Correlation between CAFRSs and stemness scores. **(I)** Heat map demonstrating the correlation between signature CAFRGs and the abundance of different types of TIICs. (ns: not significant, *P < 0.05, **P < 0.01, ***P < 0.001).

The TME landscape and immune function of the two risk groups were analyzed as indicated by the results of GSEA. The abundance of TIICs was significantly different between the high- and low-CAFRS groups. In particular, patients in the low-CAFRS group had a higher abundance of tumor-infiltrating CD8^+^ T cells and CD4^+^ T cells and a lower abundance of macrophages, especially M2 macrophages (**Figures 8A, B**). The results of the ImmunecellAI algorithm showed an increased abundance of tumor-infiltrating NK cells in the low-CAFRS group (**Figure 8B**). In addition, the low-CAFRS group had higher stromal scores, immune scores and ESTIMATE scores and lower tumor purity than the high-CAFRS group (**Figures 8C, D**). These findings indicated that low-CAFRS tumors tended to be ‘hot tumors’ with improved immune cell infiltration.

The CAFRS was significantly correlated with microenvironmental scores, with a negative correlation with immune scores and a positive correlation with tumor purity (**Figures 8E, F**). In addition, the CAFRS was positively correlated with tumor stemness scores (**Figures 8G, H**). Therefore, tumors with lower CAFRSs were characterized by better immune cell infiltration and lower tumor purity and stemness. Furthermore, a heat map was plotted to identify signature CAFRGs that were closely associated with the abundance of TIICs. The expression of CCDC8, COL12A1, CTSO, IGFBP6, MFAP4, PDLIM4, RUNX1 and TLN2 was associated with the abundance of resting mast cells and CD4^+^ T cells (**Figure 8I**). The expression of RUNX1 and COL12A1 was negatively associated with the abundance of CD8^+^ T cells, whereas that of C1S, IGFBP6, MFAP4 and PDLIM4 was significantly positively associated with the abundance of CD8^+^ T cells (**Figure 8I**). Eight CAFRGs, namely, COL12A1, CTSO, IGFBP6, MFAP4, NT5E, OSMR, RUNX1 and TLN2, were significantly associated with multiple TIICs (**Figure 8I**).

### 3.8 Discrimination power of the CAF-related risk signature for immune functional phenotypes

**Figure 9:**
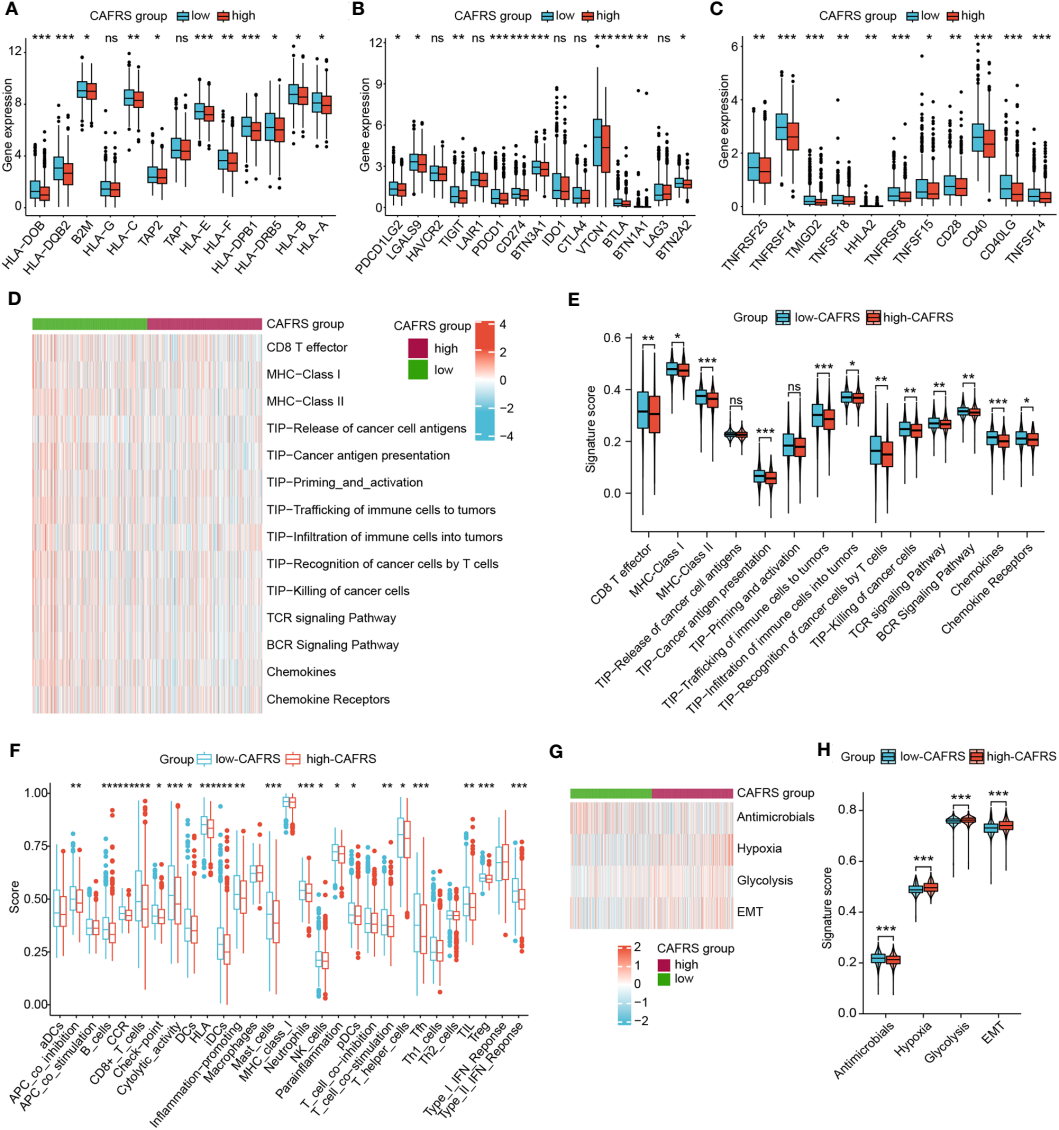
Role of the CAF-related risk signature in characterising the antitumor immunity of patients with BRCA. **(A–C)** Differential expression of antigen processing- and presentation-related genes **(A)**, inhibitory checkpoint genes **(B)** and stimulatory checkpoint genes **(C)** between the high- and low-CAFRS groups. **(D–F)** GSVA was used to assess antitumor immune function in the two CAFRS groups. **(G, H)** GSVA was used to assess cancer hallmarks in the two CAFRS groups. (ns: not significant, *P < 0.05, **P < 0.01, ***P < 0.001).

Immune function status could also affect the efficacy of tumor immunotherapy, so we explored the relationship between CAFRS and immune function phenotypes. Initially, the expression of immunomodulatory molecules was compared between the high- and low-CAFRS groups. As shown in **Figures 9A–C**, the low-CAFRS group had higher expression of antigen processing- and presentation-related genes, co-inhibitory molecules and co-stimulatory molecules. Several immunotherapeutic targets such as PDCD1 (PD-1), CD274 (PD-L1) and TIGIT were upregulated in the low-CAFRS group (**Figure 9B**). Subsequently, GSVA was performed to quantify the activities of different pathways in each sample. Apparently, low-CAFRS patients scored higher in multiple antitumor immunity-related activities such as CD8^+^ T cell effector, cancer antigen presentation, trafficking and infiltration of immune cells into tumors, T-cell mediated tumor killing, chemokines, etc. than those high-CAFRS individuals, indicating that lower CAFRSs were associated with better anti-tumor immunity (**Figures 9D–F**). As a crucial anti-tumor process, type II interferon response was significantly stronger in the low-CAFRS group (**Figure 9F**). Additionally, patients with low CAFRSs had enhanced antimicrobial potential, whereas those with high CAFRSs showed enhanced hypoxia, glycolysis, and epithelial–mesenchymal transition (EMT) activities (**Figures 9G, H**).

### 3.9 Application of CAFRS in predicting the immunotherapy response

**Figure 10:**
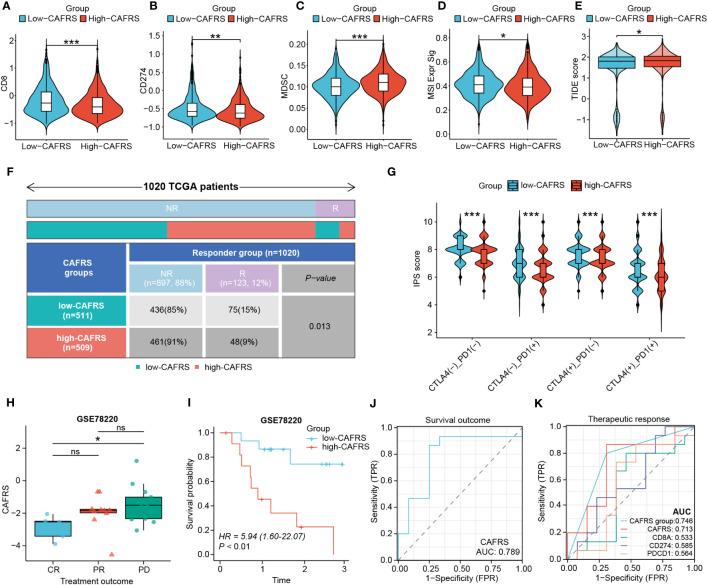
Performance of the CAF-related risk signature in predicting immunotherapy sensitivity in patients with BRCA. **(A–E)** Differences in CD8 T-cell scores **(A)**, CD274 scores **(B)**, MDSC scores **(C)**, MSI signature scores **(D)** and TIDE scores **(E)** between the high- and low-CAFRS groups. **(F)** Distribution of responders and non-responders to immunotherapy in the two CAFRS groups. **(G)** Violin plot demonstrating differences in IPSs between the two CAFRS groups. **(H–K)** Comparison of CAFRSs **(H)**, survival analysis **(I)**, ROC curve for survival outcome **(J)** and ROC curve for therapeutic response **(K)** in the external immunotherapy cohort GSE78220. (ns: not significant, *P < 0.05, **P < 0.01, ***P < 0.001).

The differential immune function between the high- and low-CAFRS groups strongly implied that patients may have varied responses to immunotherapy. The low-CAFRS group showed significantly higher CD8^+^ T-cell scores, CD274 scores, microsatellite instability (MSI) scores and TIDE scores but lower MDSC scores than the high-CAFRS group, indicating that patients with higher CAFRSs were more likely to suffer from immunosuppression and benefit less from immunotherapy (**Figures 10A–E**). As expected, the response to immunotherapy was better in the low-CAFRS group (**Figure 10F**). In addition, the low-CAFRS group had higher IPSs, which indicated a more immunogenic phenotype regardless of the PD-1 and CTLA4 status (**Figure 10G**). These results were validated in an external immunotherapy cohort. Consistently, patients with higher CAFRSs showed a poor response to immunotherapy and shorter survival (**Figures 10H, I**). The AUC values for predicting survival and therapeutic outcomes were >0.7, demonstrating the good performance of CAFRS in predicting the therapeutic response and survival of patients receiving immunotherapy (**Figures 10J, K**). Altogether, these results suggest that patients with lower CAFRSs benefit more from immunotherapy and that CAFRS is an efficient tool for predicting immunotherapy response.

### 3.10 Application of CAFRS in predicting chemotherapeutic sensitivity and identifying promising drugs

**Figure 11:**
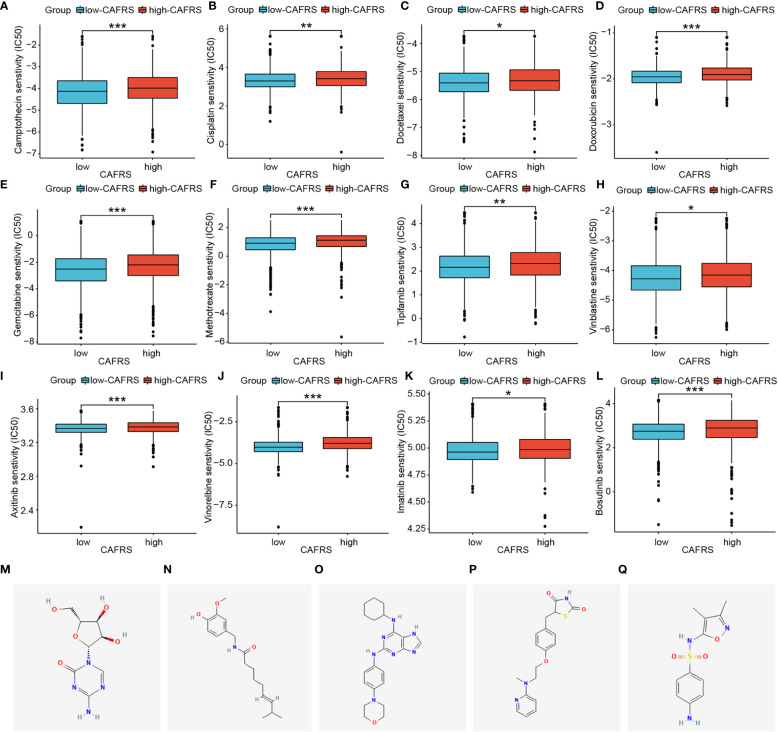
Assessment of chemotherapeutic sensitivity and prediction of candidate drugs. **(A–L)** Box plots indicate that patients with low CAFRSs tend to respond better to most chemotherapeutic and targeted drugs including camptothecin **(A)**, cisplatin **(B)**, docetaxel **(C)**, doxorubicin **(D)**, gemcitabine **(E)**, methotrexate **(F)**, tipifarnib **(G)**, vinblastine **(H)**, axitinib **(I)**, vinorelbine **(J)**, imatinib **(K)** and bosutinib **(L)**. **(M–Q)** Structures of five compounds (azacitidine, capsaicin, sulfafurazole, rosiglitazone and reversine) predicted to be promising drugs for treating patients with high CAFRSs. (*P < 0.05, **P < 0.01, ***P < 0.001).

To examine the utility of CAFRS in guiding the precise treatment of BRCA, the sensitivity of patients to several clinically common chemotherapeutic drugs, including natural, platinum-based, anti-metabolic and molecularly targeted drugs, was evaluated. Patients with high CAFRSs had elevated IC50 values for all 12 drugs selected in the analysis, implying the presence of underlying drug resistance (**Figures 11A–L**). Accordingly, patients with low CAFRSs may benefit more from these anticancer drugs, which may partly explain their better prognosis. Because high-CAFRS patients had a poor prognosis and less therapeutic benefit, targeting specific molecules is necessary for expanding therapeutic options in this population. Consequently, we analyzed the differential genes between two CAFRS subgroups and predicted the small molecule compounds promising to target high-CAFRS tumors using the Cmap platform. Azacitidine, capsaicin, sulfafurazole, rosiglitazone and reversine were identified as promising agents suitable for treating high-CAFRS patients (**Figures 11M–Q**).

### 3.11 Correlation of CAFRG-based clustering with prognosis and immunity in BRCA

Consensus clustering analysis was performed to assess the applicability of the 14 signature CAFRGs as therapeutic targets. Two stable CAF clusters were identified (A and B) based on the k-value of 2. (**Supplementary Figures 7A, B**). As shown in **Supplementary Figure 7C**, patients in cluster B had shorter overall survival than those in cluster A (*P*< 0.001). In terms of TME, the abundance of M0 and M2 macrophages was higher in cluster B, whereas that of naïve B cells, CD8^+^ T cells and gamma delta T cells was higher in cluster A (**Supplementary Figure 7D**). As shown in **Supplementary Figure 7E**, patients in cluster A with lower TIDE scores may benefit more from immunotherapy. CAF and MDSC scores were higher and MSI scores were lower in cluster B than in cluster A, indicating the immunosuppressive status and poor immunotherapeutic responsiveness of patients in cluster B (*P*< 0.001) (**Supplementary Figures 7F–H**). These results indicate that the risk signature and molecular subtypes developed based on the 14 CAFRGs are favorable tools for predicting the prognosis of BRCA.

### 3.12 Expression patterns and prognostic significance of key CAFRGs

To examine the role of signature CAFRGs in a multidimensional manner, their expression patterns were compared between BRCA and normal tissues and between patients with different tumor stages. Consequently, a total of 10 differentially expressed CAFRGs were selected for proteomic analysis (**Supplementary Figures 8A, B**). Because the expression of six CAFRGs (C1S, COL12A1, IGFBP6, MFAP4, OSMR and TLN2) was consistent at both mRNA and protein levels, they were identified as key genes for subsequent analysis (**Supplementary Figure 8C**). IHC analyses based on HPA data further validated the expression patterns of the six CAFRGs in tissue samples (**Supplementary Figures 9A, B**). In addition, survival analysis revealed that the 6 CAFRGs were robust indicators of prognosis in the external cohorts (**Supplementary Figures 9C, D**). In the single-cell dimension, the preferential expression of these six CAFRGs in CAFs was validated in two external scRNA-seq datasets (**Supplementary Figures 10A–C**, **11A–C**). This gene signature also scored higher in CAFs, illustrating its reliability (**Supplementary Figures 10D**, **S11D**). Moreover, bulk transcriptomic analyses also revealed a strong positive correlation between MFAP4 expression and the expression of common CAF markers including COL1A1, ACTA2, and FAP (**Supplementary Figures 11E**).

**Figure 12:**
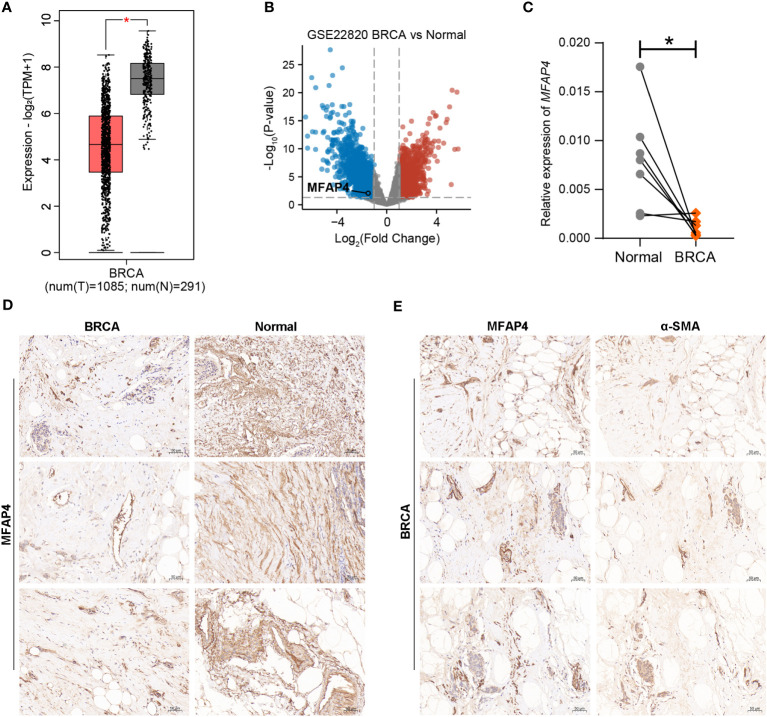
Validation of the expression pattern of MFAP4 using external datasets and experimental approaches. **(A, B)** Analyses based on the GEPIA database and external transcriptomic datasets showed that the expression of MFAP4 was upregulated in normal tissues. **(C, D)** The expression of MFAP4 in normal and BRCA tissues was determined via qRT-PCR and IHC analysis. **(E)** IHC slides showed spatial co-localization of MFAP4 with α-SMA. (*P < 0.05).

Among the 6 key CAFRGs, MFAP4 was selected for subsequent analysis owing to its under-reported status in BRCA. The expression of MFAP4 was found to be lower in BRCA tissues than in normal tissues in multiple datasets, and our IHC and qRT-PCR results consistently confirmed this finding (**Figures 12A–D**). In addition, MFAP4 exhibited strong co-localization with CAFs in BRCA samples (**Figure 12E**).

## 4 Discussion

BRCA is the leading cause of cancer-related death among women and the most common malignancy worldwide (1). However, effective treatment strategies for advanced or metastatic BRCA are still limited (2). Recently, numerous clinical trials have shown that immunotherapy, represented by immune checkpoint inhibitors, may have improved therapeutic effects against cancer, especially middle- and advanced-stage cancer when combined with other therapies (6–8). With in-depth research on immunotherapy, scholars are increasingly focusing on the role of TME in influencing prognosis and antitumor immunity (9, 40). As an important component of TME, CAFs are involved in the remodeling of the extracellular matrix (ECM) and secrete various cytokines, chemokines, and pro-angiogenic factors (41). They actively participate in the continuous interactions among cancer cells, endothelial cells and immune cells in the hypoxic TME, which may lead to immunosuppression (12). For example, a CAF-derived secreted protein, Biglycan (BGN), was found to be negatively correlated with CD8^+^ T cell infiltration, and its high expression indicated poor prognosis and an immunosuppressive microenvironment in BRCA (42). In addition, CAFs may promote the progression of BRCA by inducing angiogenesis and recruiting bone marrow-derived endothelial cells (16). Recent studies have reported that CAFs are involved in the development of resistance to chemotherapy (43).

The abovementioned functions of CAFs indicate their therapeutic potential in cancer. For example, therapeutic approaches based on tumor vaccines or chimeric antigen receptor (CAR) T-cell therapies targeting fibroblast activation protein (FAP), a surface marker of CAF, have been shown to reactivate antitumor immune responses and inhibit tumor progression (44, 45). Reversing CAF-mediated ECM remodeling can promote the function of immune effector cells (46). However, therapies targeting CAFs are limited owing to the lack of effective biomarkers (10). CAF-related prognostic signatures with favorable predictive performance in BRCA remain underreported and lack the integration of single-cell and bulk transcriptomic data as well as the potential for assessing treatment responses. Therefore, novel valuable CAF-specific targets should be identified and their significance in assessing prognosis and therapeutic responses in BRCA should be investigated intensively.

In this study, our goal is to uncover CAF-associated prognostic genes using reliable screening strategies, based on which we will establish novel CAF-associated prognostic biomarkers to provide new targets and ideas for precision medicine of BRCA. We first analyzed the effect of CAF levels on the prognosis of BRCA patients, which showed that patients with more infiltrative CAFs had a poorer prognosis, indicating the significance of investigating the role of CAFRGs in BRCA. Consequently, two-step bulk transcriptomic WGCNA and scRNA-seq analyses were integrated to develop a CAF-related risk signature for BRCA. Patients with BRCA were effectively stratified using this signature, and its favorable performance in predicting prognosis, clinicopathologic features, immune landscapes, and therapeutic responsiveness was verified in multiple BRCA datasets. High-CAFRS patients had worse clinical outcomes, significant TME immunosuppression and poor therapeutic responses. In addition, a real-world immunotherapy cohort also validated the predictive results. These findings may provide a theoretical reference for the further application of the CAF-based risk signature in BRCA.

Another important finding of this study is the identification of novel promising CAF-related therapeutic targets for BRCA. The 14 CAFRGs (C1s, CCDC8, COL12A1, CTSO, IGFBP6, MFAP4, NT5E, OSMR, PDLIM4, RUNX1, SAV1, SDC1, SGCE and TLN2) involved in the risk signature were identified as potential prognostic biomarkers strongly associated with TME and therapeutic effectiveness. Complement is an important member involved in inflammation and immunity, and activation of complement C1 has been associated with the malignant phenotype and poor prognosis of cancer (47, 48). In addition, complement C1q and C1s are involved in the formation of an immunosuppressive TME (49). Insulin-like growth factor-binding protein 6 (IGFBP6) plays a role in promoting angiogenesis and metastasis as a result of IGF-independent effects (50). RUNX family transcription factor 1 (RUNX1), a tumor suppressor, and RUNX2 have contradictory regulatory effects on EMT in BRCA. Downregulation of RUNX1 and upregulation of RUNX2 drive EMT in early-stage BRCA (51). TLN2 encoding talin2 plays an important role in cancer metastasis by regulating the traction force, focal adhesion and invadopodia (52). PDZ and LIM domain 4 (PDLIM4), also known as RIL, is thought to be a suppressor of ovarian, breast and prostate cancers, and its low expression has been associated with hypermethylation in both primary BRCA and lymph node metastases (53–55). Dysregulation of CCDC8 occurs in the early stage of BRCA progression and is associated with tumor metastasis to the brain and other organs (56). COL12A1 has been reported as a poor prognostic indicator for cancers including BRCA, gastric cancer (GC) and cholangiocarcinoma (57–59). Recently, a study described the mechanism of COL12A1 in facilitating the generation of a pro-invasive TME conducive to the metastasis of BRCA (57). Its family member, COL11A1 was also identified to be a CAF-associated prognostic predictor in BRCA by an integrated machine learning approach (38). As another CAFRG with repressed expression resulting from epigenetic mechanisms in BRCA, MFAP4 has been functionally associated with the damage and alterations of elastic fibers and its high expression has been reported to indicate a better prognosis in BRCA (60). SGCE regulates the accumulation and remodeling of the ECM, and its knockdown inhibits the self-renewal ability, metastasis and drug resistance of BRCA stem cells (61). Oncostatin M (OSM)/oncostatin M receptor (OSMR) signaling regulates the interactions among CAFs, cancer cells and immune cells, thereby reprogramming the pro-tumorigenic and pro-metastatic TME (14, 62). Syndecan-1 (SDC1), a member of the syndecan family, regulates the invasive behavior of BRCA cells, and its upregulation is associated with a poorer prognosis, more advanced clinical stage and more malignant phenotypes in BRCA (63, 64). Ecto-5´-nucleotidase (NT5E), also known as CD73, is upregulated in metastatic BRCA and participates in facilitating a poor prognosis, chemotherapeutic resistance and immunosuppression (65–68). CTSO is a cysteine protease that is involved in the selective activation of macrophage-mediated matrix remodeling and osteoclast-mediated bone resorption (69). Apart from these consistent findings, we have further raised the underlying interactions of these CAFRGs with tumor-infiltrating immune cells by gene-cell correlation analysis. Certain CAFRGs included in the risk signature have been reported to affect the progression and prognosis of BRCA, which partially indicates the reliability of the risk signature and therapeutic targets screened in this study. The potential therapeutic value of the 14 CAFRGs warrants further investigation.

Altogether, we have proposed a new CAF-associated prognostic gene signature for breast cancer in this study; in terms of methodology, a novel approach of combining bulk sequencing with single-cell sequencing to screen genes was implemented; regarding the prognostic performance, the signature performed well in terms of survival prediction, clinicopathological relevance, and therapeutic responsiveness prediction; the key gene MFAP4 was screened, and its expression and prognostic features were characterized and validated using external datasets and experimental approaches. However, this study also has some shortcomings. The application value of this prognostic model needs to be explored in clinical practice. In addition, the biological functions of the CAFRGs identified in this study require more in-depth experiments for validation. Nevertheless, this study provides a theoretical and preliminary basis for the identification of novel CAFRGs that may facilitate the individualized assessment and management of patients with BRCA.

## 5 Conclusions

The CAF-derived risk signature developed in this study is a reliable tool for predicting the prognosis, immune characteristics, and treatment response of patients with BRCA. In addition, this study provides valuable insights into the mechanisms underlying the progression of BRCA and proposes novel therapeutic targets for BRCA.

## Data availability statement

The datasets presented in this study can be found in online repositories. The names of the repository/repositories and accession number(s) can be found in the article/**Supplementary Material**.

## Ethics statement

The studies involving humans were approved by the Ethics Committee of Changhai Hospital, Naval Medical University. The studies were conducted in accordance with the local legislation and institutional requirements. The participants provided their written informed consent to participate in this study. Written informed consent was obtained from the individual(s) for the publication of any potentially identifiable images or data included in this article.

## Author contributions

CL: Data curation, Methodology, Software, Visualization, Writing – original draft, Writing – review & editing. LY: Data curation, Methodology, Software, Writing – original draft, Visualization, Writing – review & editing. YZ: Data curation, Formal analysis, Software, Validation, Writing – review & editing, Writing – original draft. QH: Data curation, Formal analysis, Writing – review & editing. SW: Data curation, Formal analysis, Writing – review & editing. SL: Formal analysis, Investigation, Writing – review & editing. YT: Conceptualization, Investigation, Supervision, Validation, Writing – review & editing. WH: Conceptualization, Data curation, Investigation, Supervision, Validation, Writing – review & editing. LZ: Conceptualization, Data curation, Formal analysis, Funding acquisition, Investigation, Methodology, Project administration, Supervision, Writing – review & editing.

## Supplementary material

Supplementary Table 1 Primer sequences used in experiments.

Supplementary Table 2 List of 43 prognostic CAFRGs.

Supplementary Figure 1 WGCNA was performed to cluster samples and assess the correlation between modular genes and CAF infiltration. **(A, C)** Samples were clustered and those with branch positions above the red line were removed. **(B, D)** Modular genes were strongly positively correlated with CAF infiltration. The results displayed on the left panel **(A, B)** are based on the MCPcounter algorithm, and those displayed on the right panel **(C, D)** are based on the xCELL algorithm.

Supplementary Figure 2 Feature plots **(A)** and heat map **(B)** demonstrating the differential expression of marker genes among different cell types.

Supplementary Figure 3 Assessment of the independent prognostic potential of the CAF-related risk signature. **(A, B)** Assessment of the independent prognostic potential of the CAF-related risk signature in TCGA-BRCA cohort. **(C, D)** Validation of the independent prognostic potential of the CAF-related risk signature in two external cohorts.

Supplementary Figure 4 Verification of the clinicopathological relevance of the CAF-related risk signature in two external cohorts. **(A, B)** ROC curves demonstrating the predictive performance of the signature. **(C, D)** Heat maps of patients with different clinicopathological characteristics in the high- and low-CAFRS groups. **(E–I)** Histograms and box plots demonstrating the relationship between the CAFRS and clinicopathological parameters including age **(E)**, grade **(F)**, tumor size **(G)**, survival status **(H)** and positive lymph node (LN^+^) status **(I)** in the GSE96058 cohort. **(J, K)** Histograms and box plots demonstrating the relationship between the CAFRS and clinicopathological parameters including positive lymph node (LN^+^) status **(J)** and age **(K)** in the METABRIC cohort. **(L)** RFS analysis in the METABRIC cohort. The results shown in **(B, D, J-L)** are based on the METABRIC cohort, and those shown in **(A, C, E–I)** are based on the GSE96058 cohort.

Supplementary Figure 5 Survival analyses in different clinical subgroups to test the stability of the signature. **(A)** Survival analyses conducted in the TCGA-BRCA cohort. **(B)** Survival analyses conducted in the GSE96058 cohort.

Supplementary Figure 6 Gene set enrichment analysis based on HALLMARK pathways. Significantly enriched HALLMARK pathways in the high-CAFRS **(A)** and low-CAFRS **(B)** groups.

Supplementary Figure 7 Consensus clustering analysis based on 14 signature CAFRGs. **(A, B)** Based on the k value of 2, samples in the TCGA-BRCA cohort were divided into two CAF clusters. **(C)** Patients in CAF cluster B experienced shorter survival than those in CAF cluster A. **(D–H)** Differences in the abundance of TIICs **(D)**, TIDE scores **(E)**, MDSC signature scores **(F)**, CAF signature scores **(G)** and MSI signature scores **(H)** between the two CAF clusters.

Supplementary Figure 8 Selection of key CAFRGS. **(A, B)** Differential expression analyses in tissues and clinicopathologic subgroups. **(C)** Proteomic expression of CAFRGs based on the CPTAC database.

Supplementary Figure 9 Validation of the protein levels and prognostic roles of six key CAFRGs. **(A, B)** Protein expression of the six key CAFRGs based on IHC images from the HPA database. **(C, D)** Survival analysis of the six key CAFRGs in external datasets.

Supplementary Figure 10 Validation of the expression profiles of six key CAFRGs in the external scRNA-seq dataset GSE176078. **(A)** Annotated cell types. **(B, C)** Differential expression of the six key CAFRGs in different cell types. **(D, E)** Distribution of CAFRSs in different cell types.

Supplementary Figure 11 Validation of the expression profiles of six key CAFRGs in the external scRNA-seq dataset GSE114727. **(A)** Annotated cell types. **(B, C)** Differential expression of the six key CAFRGs in different cell types. **(D, E)** Distribution of CAFRSs in different cell types. **(E)** Correlation analysis between the key gene MFAP4 and classical CAF markers.
