# Supplementary material for: Integrating single-cell and bulk transcriptomic analyses to develop a cancer-associated fibroblast-derived biomarker for predicting prognosis and therapeutic response in breast cancer
Source: Front Immunol. 2024 Jan 3;14:1307588. doi: 10.3389/fimmu.2023.1307588 (PMC10791883; doi:10.3389/fimmu.2023.1307588)
Supplement: Supplementary Table 1 — Primer sequences used in experiments. [file Table_1.docx]

**SUPPLEMENTARY TABLE S1. Primer sequences used in experiments.**

| **Species** | **Name** | **Application** | **Sequence** |
| --- | --- | --- | --- |
| Human | MFAP4-F | Primer for qRT-PCR | TACCAGTCAGACGGCGTGTA |
| Human | MFAP4-R | Primer for qRT-PCR | CCACTCGCAGCTCATACTTCT |
| Human | GAPDH-F | Primer for qRT-PCR | GACTTCAACAGCAACTCCCAC |
| Human | GAPDH-R | Primer for qRT-PCR | TCCACCACCCTGTTGCTGTA |

**SUPPLEMENTARY TABLE S2. List of 43 prognostic CAFRGs.**

| **Symbol** | **Hazard ratio (HR)** | **95% CI of HR** | ***P*-value** |
| --- | --- | --- | --- |
| H6PD | 0.687 | 0.498–0.948 | 0.022 |
| FXYD6 | 0.620 | 0.447–0.860 | 0.004 |
| CLMP | 0.695 | 0.503–0.960 | 0.027 |
| SPARCL1 | 0.710 | 0.514–0.983 | 0.039 |
| TCF7L2 | 0.720 | 0.523–0.991 | 0.044 |
| TGFBR1 | 1.586 | 1.148–2.192 | 0.005 |
| SH3BP5 | 0.693 | 0.503–0.956 | 0.026 |
| DST | 0.688 | 0.498–0.950 | 0.023 |
| SYNPO2 | 0.707 | 0.511–0.979 | 0.037 |
| CDKN1C | 0.725 | 0.527–0.998 | 0.049 |
| ENC1 | 1.467 | 1.064–2.023 | 0.019 |
| PLPP3 | 0.700 | 0.506–0.968 | 0.031 |
| DNM1 | 0.704 | 0.509–0.973 | 0.033 |
| PTN | 0.691 | 0.501–0.954 | 0.025 |
| CCDC8 | 0.606 | 0.437–0.840 | 0.003 |
| TLN2 | 0.579 | 0.416–0.806 | 0.001 |
| NT5E | 1.426 | 1.034–1.968 | 0.031 |
| COL11A1 | 1.408 | 1.023–1.938 | 0.036 |
| OSMR | 0.687 | 0.499–0.948 | 0.022 |
| SDC1 | 1.459 | 1.060–2.009 | 0.021 |
| CHPF | 1.582 | 1.148–2.179 | 0.005 |
| TSHZ2 | 0.703 | 0.507–0.976 | 0.035 |
| COL12A1 | 1.397 | 1.011–1.928 | 0.042 |
| SUGCT | 1.399 | 1.017–1.925 | 0.039 |
| CYP1B1 | 0.718 | 0.521–0.990 | 0.043 |
| SLC40A1 | 0.710 | 0.516–0.977 | 0.036 |
| CTSO | 0.717 | 0.518–0.991 | 0.044 |
| EDIL3 | 1.402 | 1.018–1.930 | 0.039 |
| RUNX1 | 0.625 | 0.451–0.864 | 0.004 |
| PTPRK | 1.381 | 1.001–1.904 | 0.049 |
| C1S | 0.669 | 0.483–0.927 | 0.016 |
| BACE2 | 1.431 | 1.036–1.976 | 0.030 |
| MFAP4 | 0.688 | 0.499–0.949 | 0.023 |
| LARP6 | 0.672 | 0.487–0.926 | 0.015 |
| PROS1 | 0.700 | 0.505–0.969 | 0.032 |
| GRP | 0.710 | 0.513–0.983 | 0.039 |
| BOC | 0.677 | 0.491–0.934 | 0.018 |
| SPECC1 | 1.484 | 1.076–2.047 | 0.016 |
| SAV1 | 0.650 | 0.472–0.896 | 0.008 |
| PDLIM4 | 0.658 | 0.476–0.908 | 0.011 |
| IGFBP6 | 0.679 | 0.491–0.939 | 0.019 |
| SGCE | 0.720 | 0.523–0.993 | 0.045 |
| IGFBP4 | 0.706 | 0.512–0.973 | 0.033 |
