# Supplementary figures and images for: Integrating single-cell and bulk transcriptomic analyses to develop a cancer-associated fibroblast-derived biomarker for predicting prognosis and therapeutic response in breast cancer
Source: Front Immunol. 2024 Jan 3;14:1307588. doi: 10.3389/fimmu.2023.1307588 (PMC10791883; doi:10.3389/fimmu.2023.1307588)

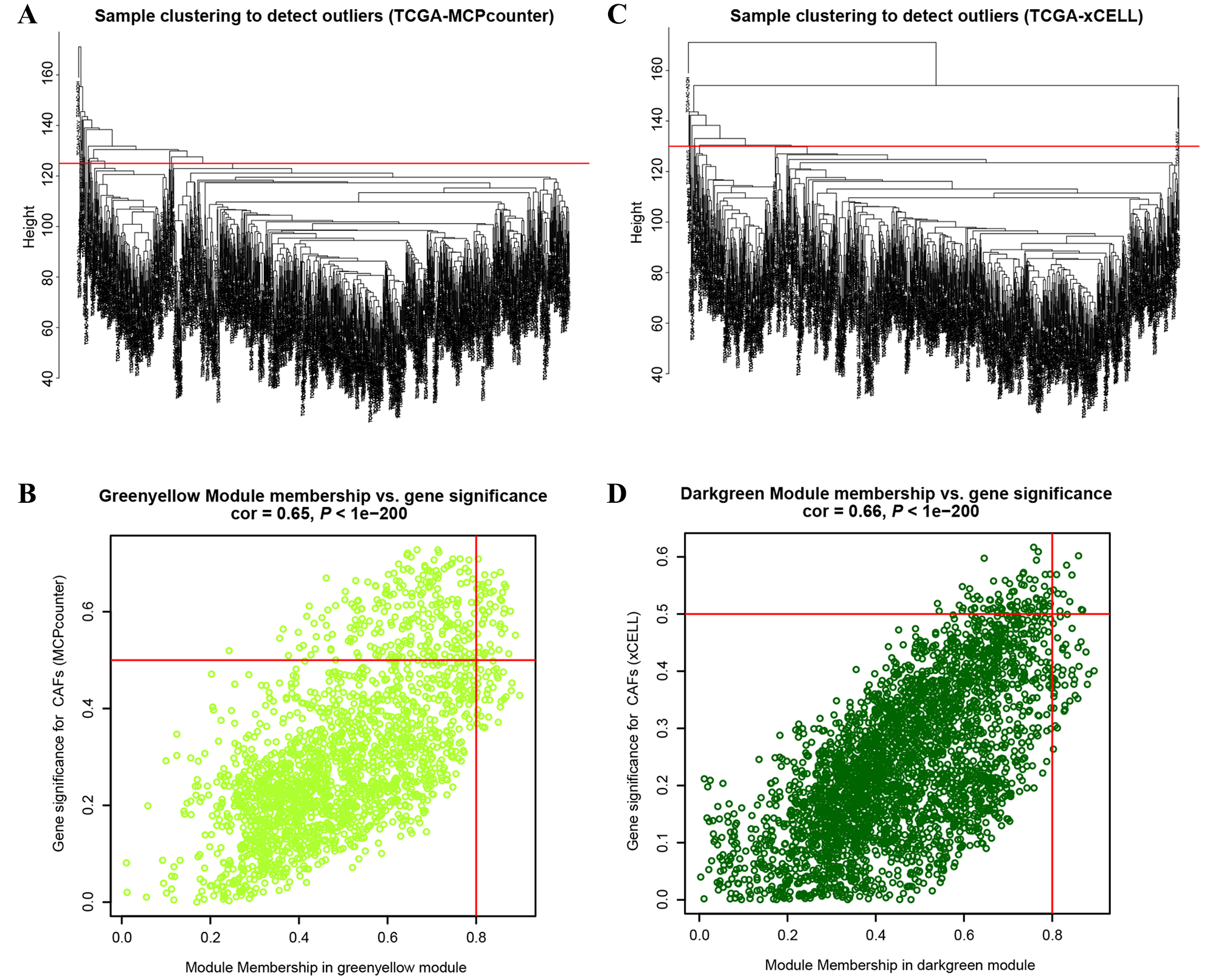

Supplement: Supplementary Figure 1 — WGCNA was performed to cluster samples and assess the correlation between modular genes and CAF infiltration. (A, C) Samples were clustered and those with branch positions above the red line were removed. (B, D) Modular genes were strongly positively correlated with CAF infiltration. The results displayed on the left panel (A, B) are based on the MCPcounter algorithm, and those displayed on the right panel (C, D) are based on the xCELL algorithm. [file DataSheet_1.zip › Supplementary Figure S1.tif]

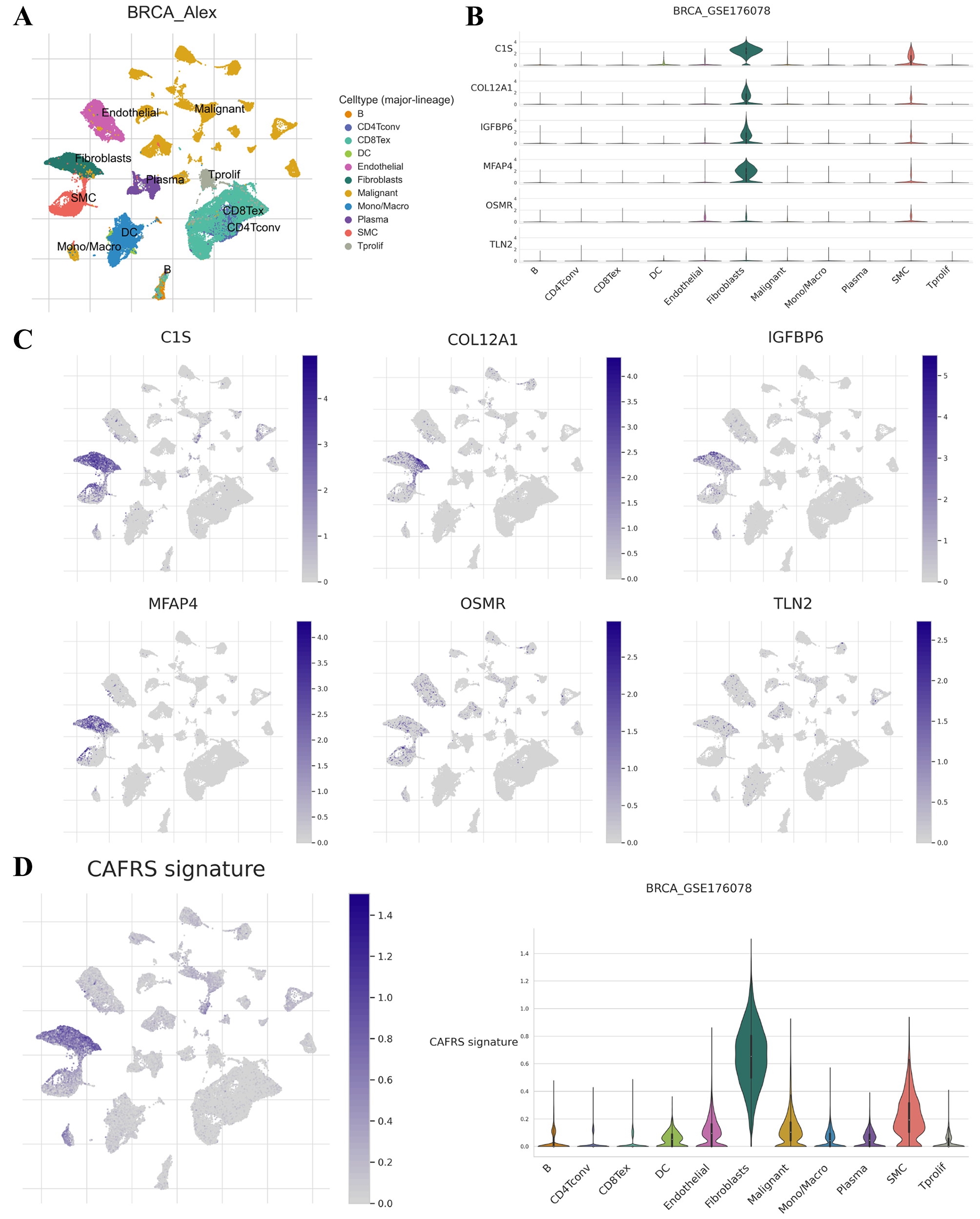

Supplement: Supplementary Figure 1 — WGCNA was performed to cluster samples and assess the correlation between modular genes and CAF infiltration. (A, C) Samples were clustered and those with branch positions above the red line were removed. (B, D) Modular genes were strongly positively correlated with CAF infiltration. The results displayed on the left panel (A, B) are based on the MCPcounter algorithm, and those displayed on the right panel (C, D) are based on the xCELL algorithm. [file DataSheet_1.zip › Supplementary Figure S10.tif]

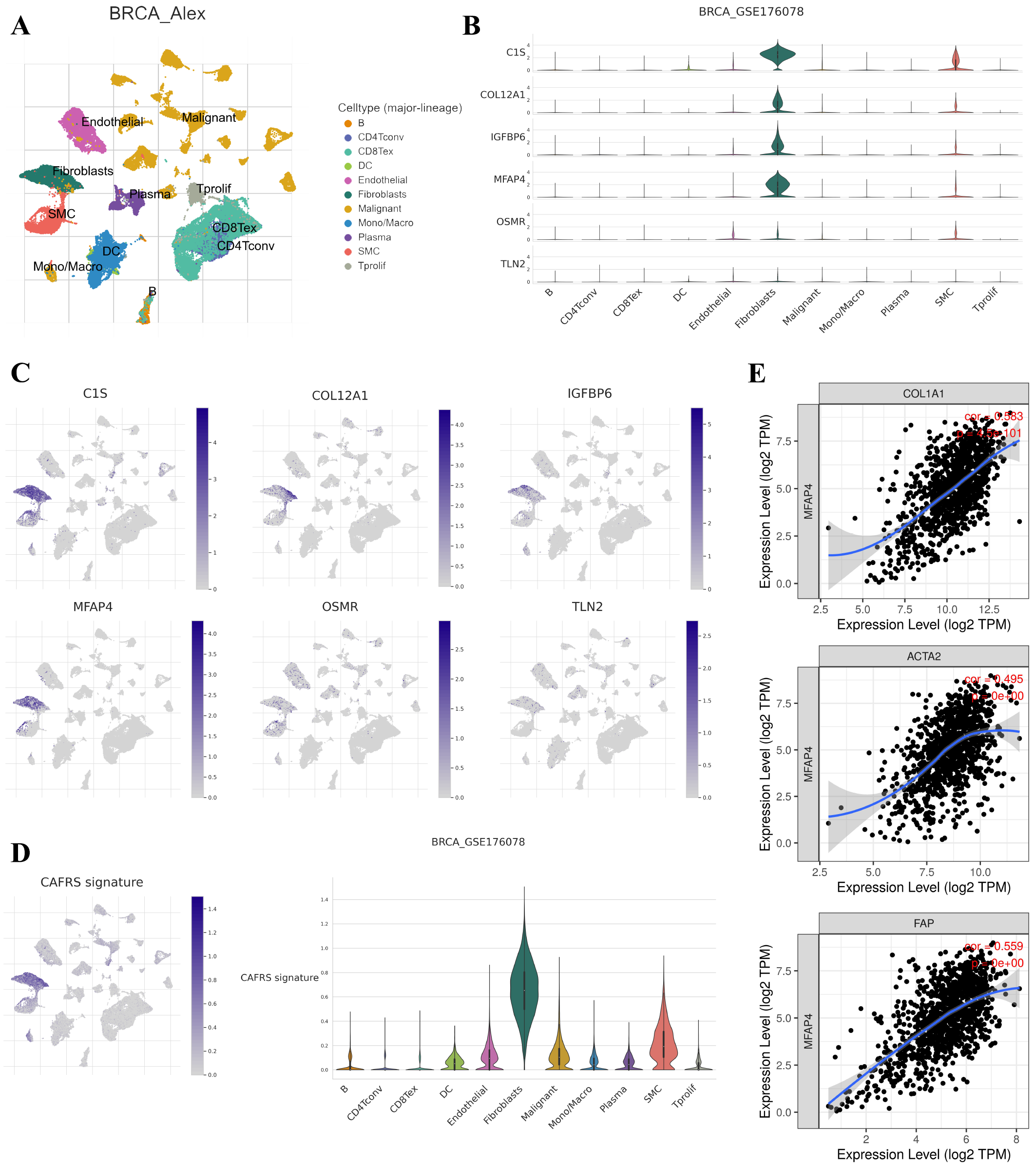

Supplement: Supplementary Figure 1 — WGCNA was performed to cluster samples and assess the correlation between modular genes and CAF infiltration. (A, C) Samples were clustered and those with branch positions above the red line were removed. (B, D) Modular genes were strongly positively correlated with CAF infiltration. The results displayed on the left panel (A, B) are based on the MCPcounter algorithm, and those displayed on the right panel (C, D) are based on the xCELL algorithm. [file DataSheet_1.zip › Supplementary Figure S11.tif]

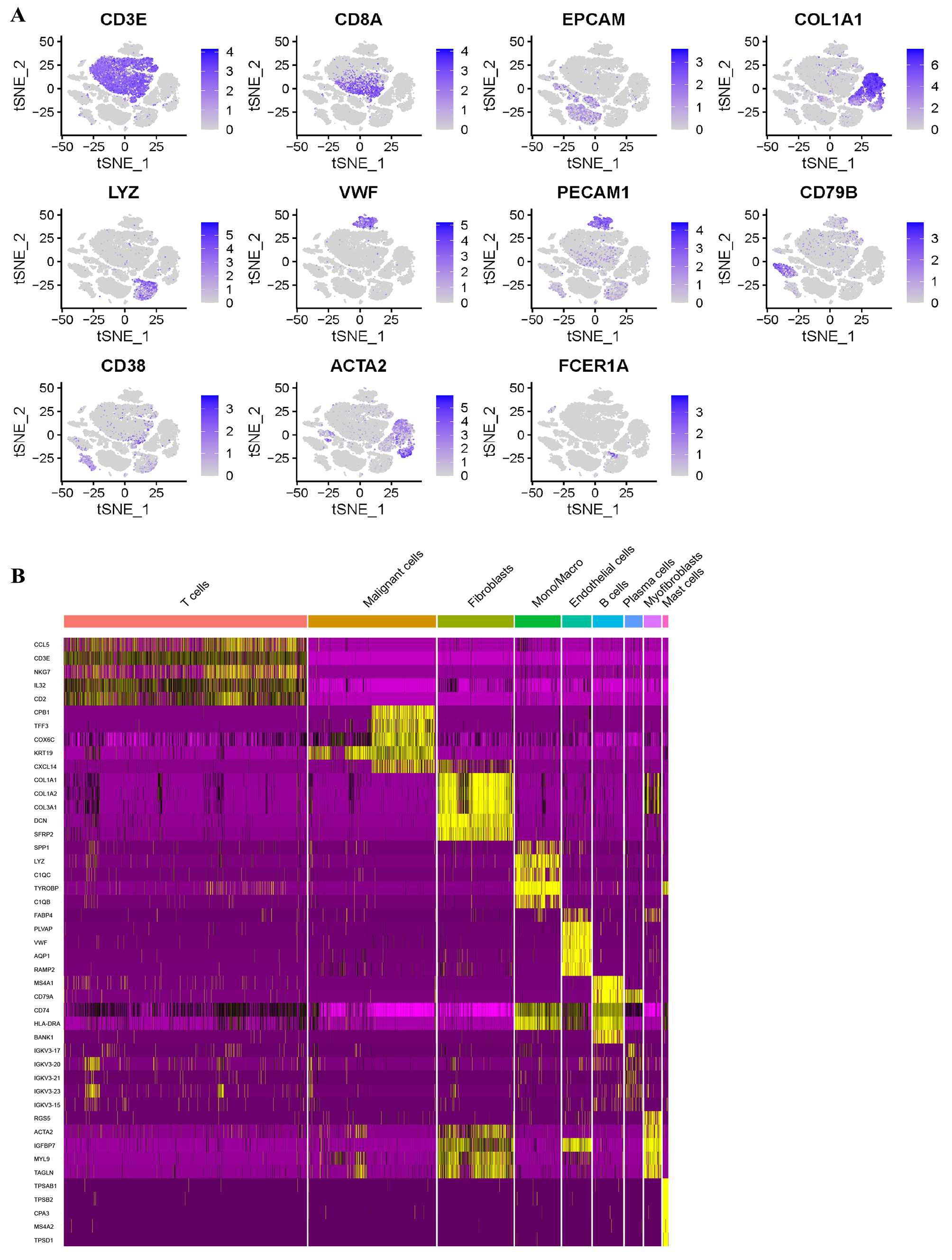

Supplement: Supplementary Figure 1 — WGCNA was performed to cluster samples and assess the correlation between modular genes and CAF infiltration. (A, C) Samples were clustered and those with branch positions above the red line were removed. (B, D) Modular genes were strongly positively correlated with CAF infiltration. The results displayed on the left panel (A, B) are based on the MCPcounter algorithm, and those displayed on the right panel (C, D) are based on the xCELL algorithm. [file DataSheet_1.zip › Supplementary Figure S2.tif]

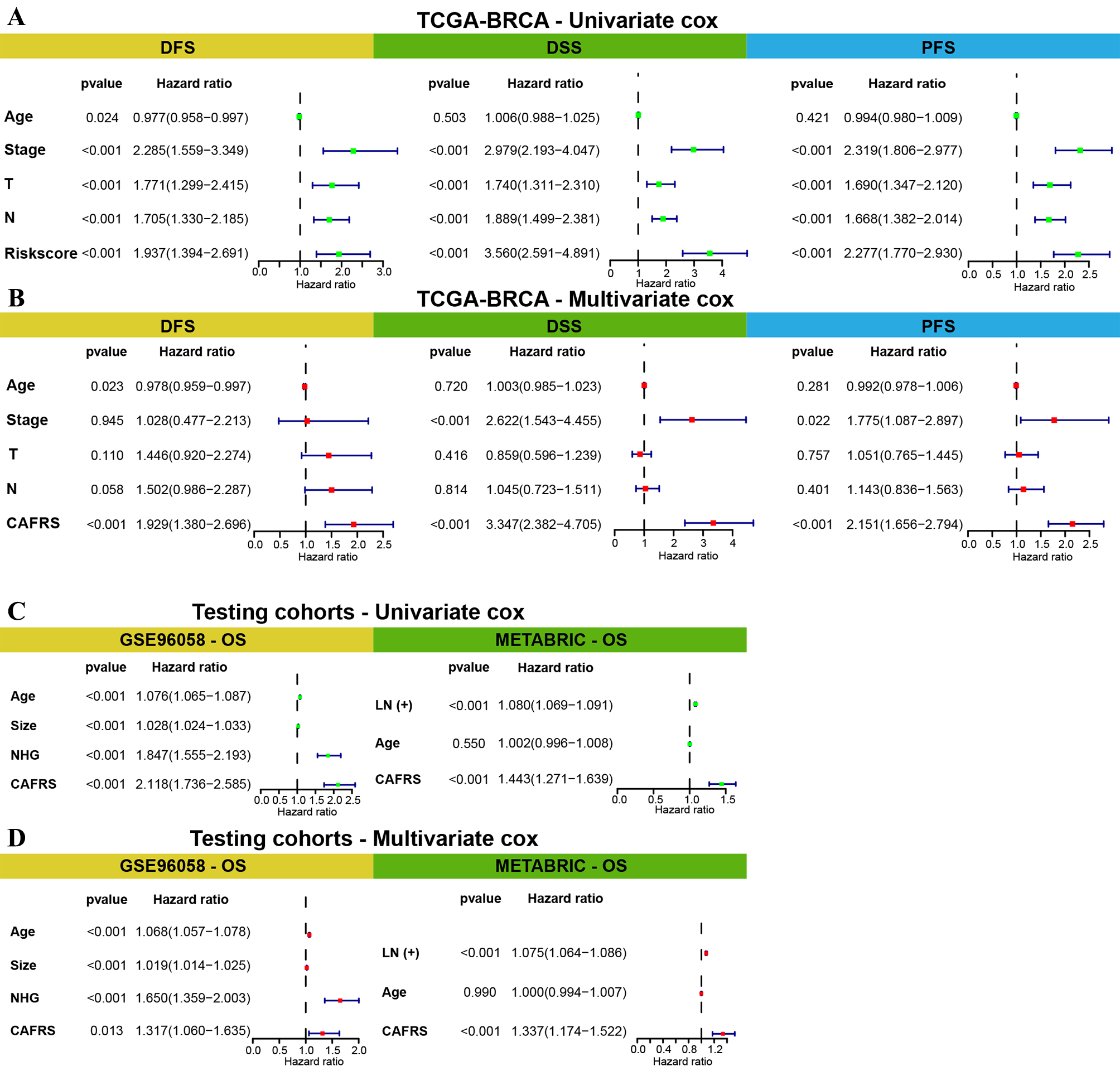

Supplement: Supplementary Figure 1 — WGCNA was performed to cluster samples and assess the correlation between modular genes and CAF infiltration. (A, C) Samples were clustered and those with branch positions above the red line were removed. (B, D) Modular genes were strongly positively correlated with CAF infiltration. The results displayed on the left panel (A, B) are based on the MCPcounter algorithm, and those displayed on the right panel (C, D) are based on the xCELL algorithm. [file DataSheet_1.zip › Supplementary Figure S3.tif]

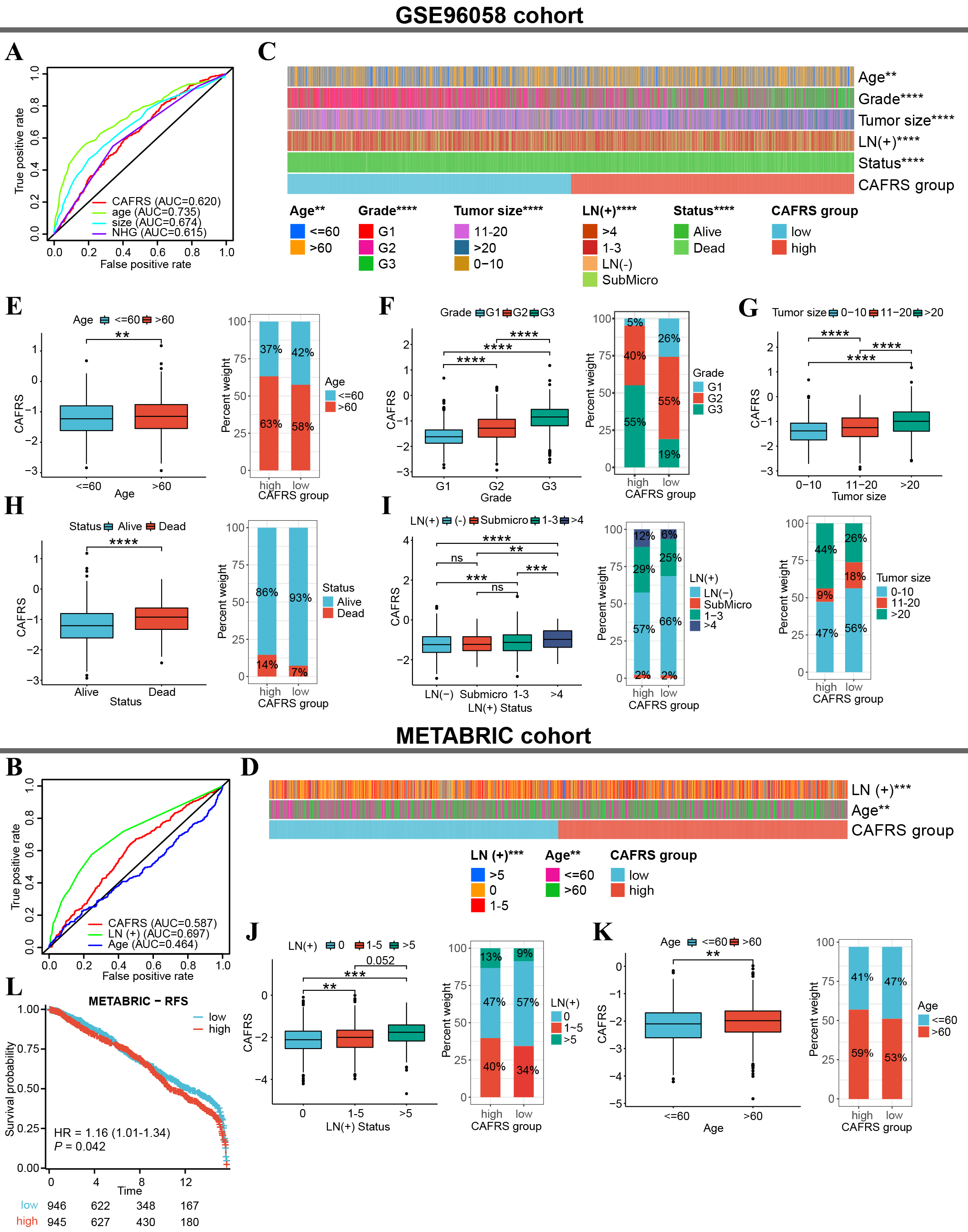

Supplement: Supplementary Figure 1 — WGCNA was performed to cluster samples and assess the correlation between modular genes and CAF infiltration. (A, C) Samples were clustered and those with branch positions above the red line were removed. (B, D) Modular genes were strongly positively correlated with CAF infiltration. The results displayed on the left panel (A, B) are based on the MCPcounter algorithm, and those displayed on the right panel (C, D) are based on the xCELL algorithm. [file DataSheet_1.zip › Supplementary Figure S4.tif]

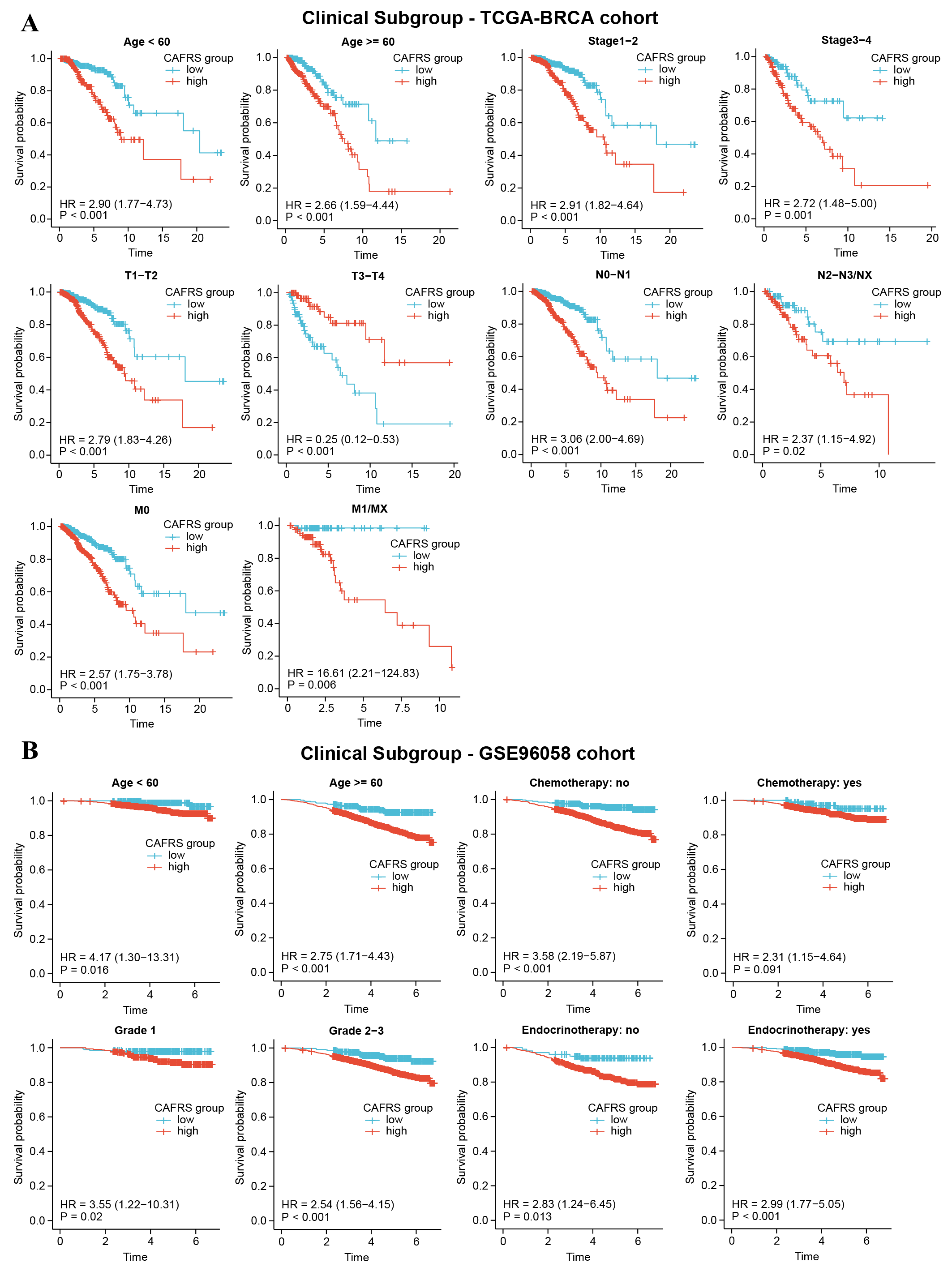

Supplement: Supplementary Figure 1 — WGCNA was performed to cluster samples and assess the correlation between modular genes and CAF infiltration. (A, C) Samples were clustered and those with branch positions above the red line were removed. (B, D) Modular genes were strongly positively correlated with CAF infiltration. The results displayed on the left panel (A, B) are based on the MCPcounter algorithm, and those displayed on the right panel (C, D) are based on the xCELL algorithm. [file DataSheet_1.zip › Supplementary Figure S5.tif]

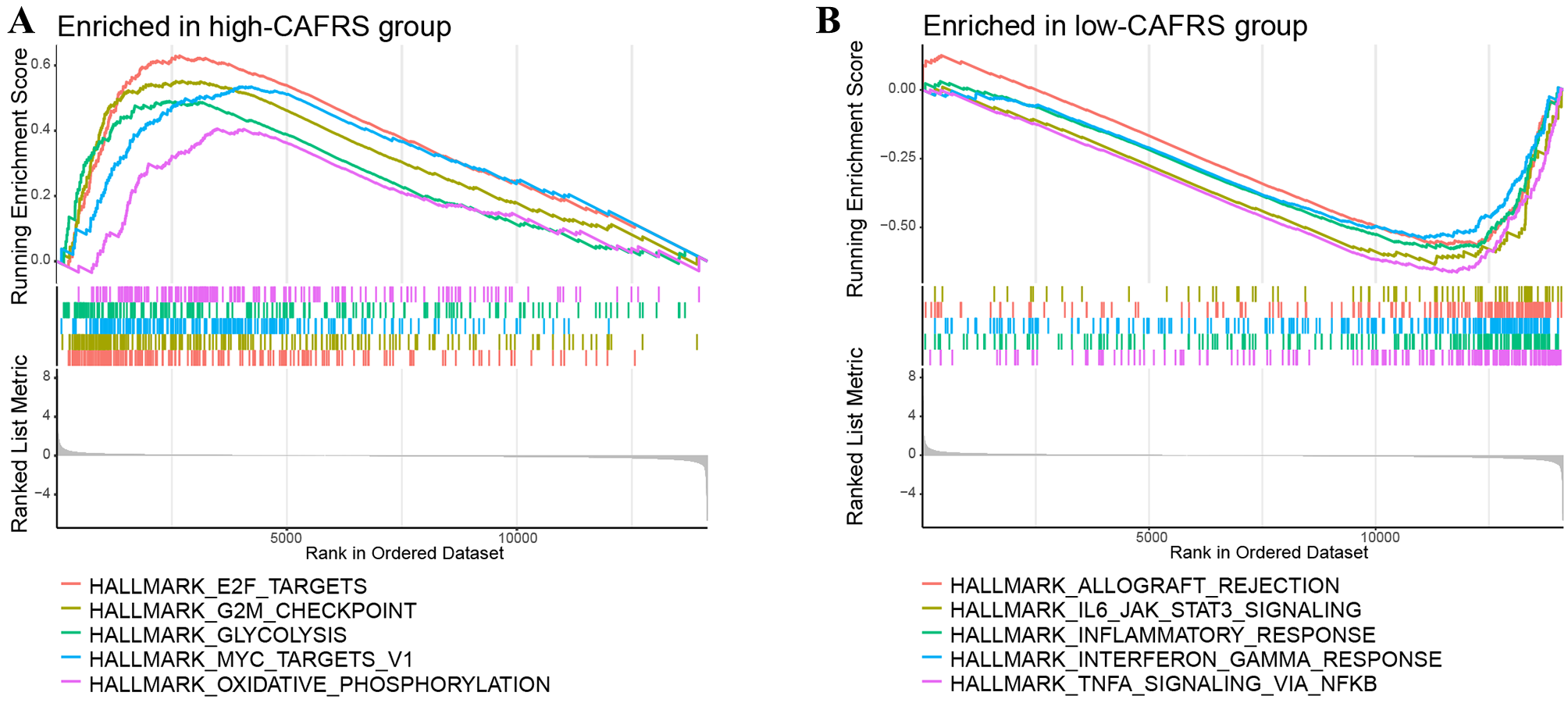

Supplement: Supplementary Figure 1 — WGCNA was performed to cluster samples and assess the correlation between modular genes and CAF infiltration. (A, C) Samples were clustered and those with branch positions above the red line were removed. (B, D) Modular genes were strongly positively correlated with CAF infiltration. The results displayed on the left panel (A, B) are based on the MCPcounter algorithm, and those displayed on the right panel (C, D) are based on the xCELL algorithm. [file DataSheet_1.zip › Supplementary Figure S6.tif]

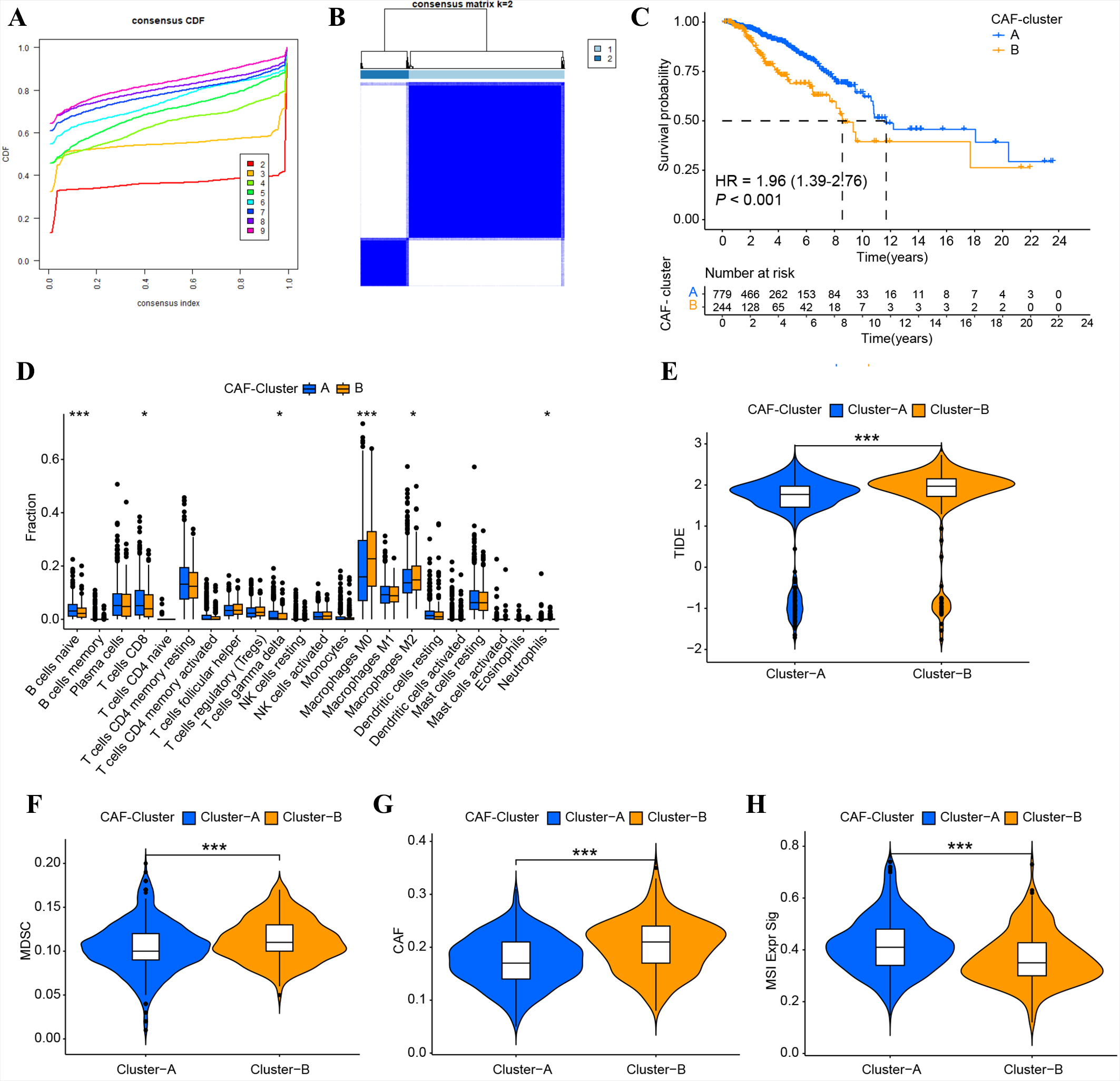

Supplement: Supplementary Figure 1 — WGCNA was performed to cluster samples and assess the correlation between modular genes and CAF infiltration. (A, C) Samples were clustered and those with branch positions above the red line were removed. (B, D) Modular genes were strongly positively correlated with CAF infiltration. The results displayed on the left panel (A, B) are based on the MCPcounter algorithm, and those displayed on the right panel (C, D) are based on the xCELL algorithm. [file DataSheet_1.zip › Supplementary Figure S7.tif]

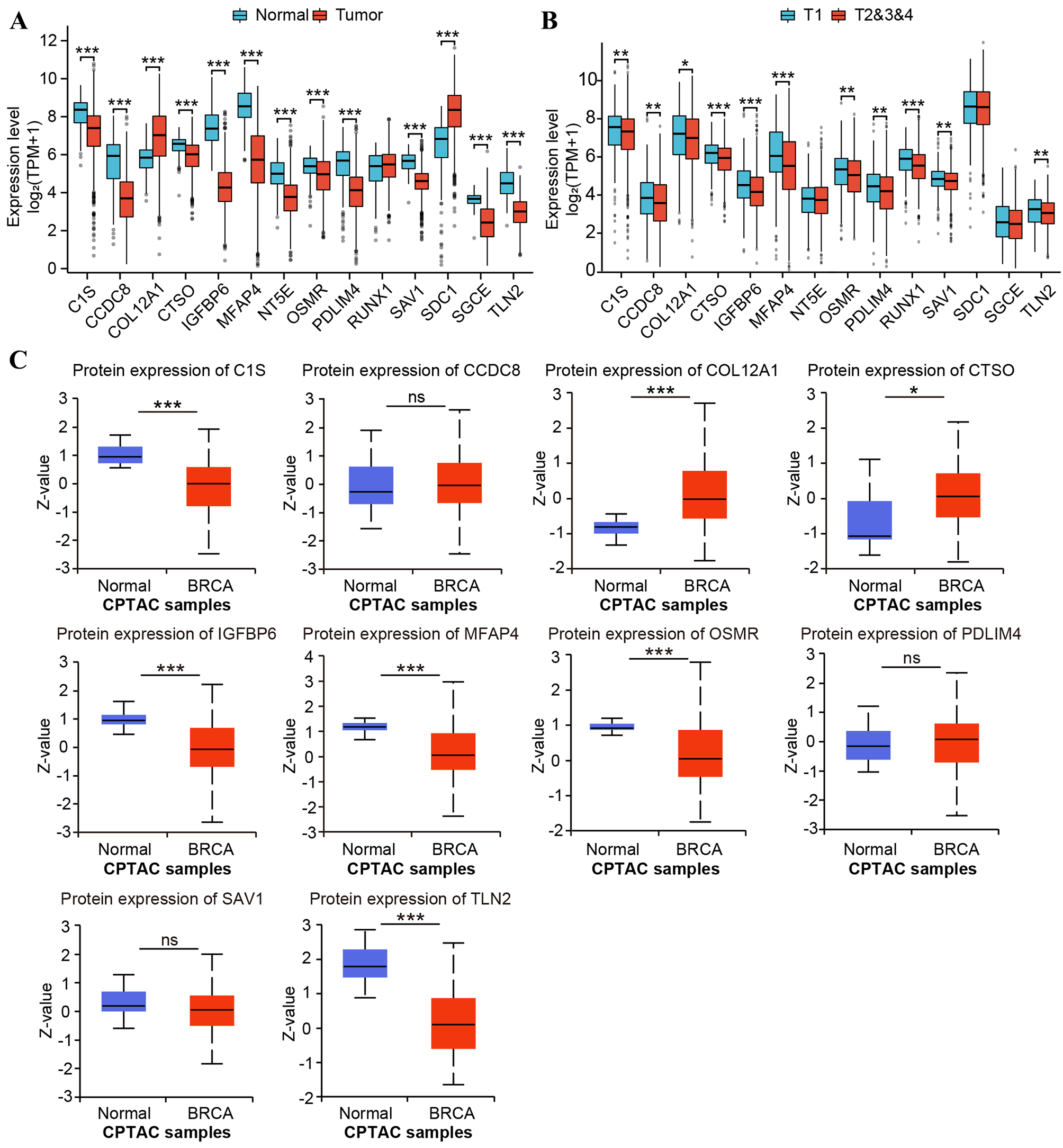

Supplement: Supplementary Figure 1 — WGCNA was performed to cluster samples and assess the correlation between modular genes and CAF infiltration. (A, C) Samples were clustered and those with branch positions above the red line were removed. (B, D) Modular genes were strongly positively correlated with CAF infiltration. The results displayed on the left panel (A, B) are based on the MCPcounter algorithm, and those displayed on the right panel (C, D) are based on the xCELL algorithm. [file DataSheet_1.zip › Supplementary Figure S8.tif]

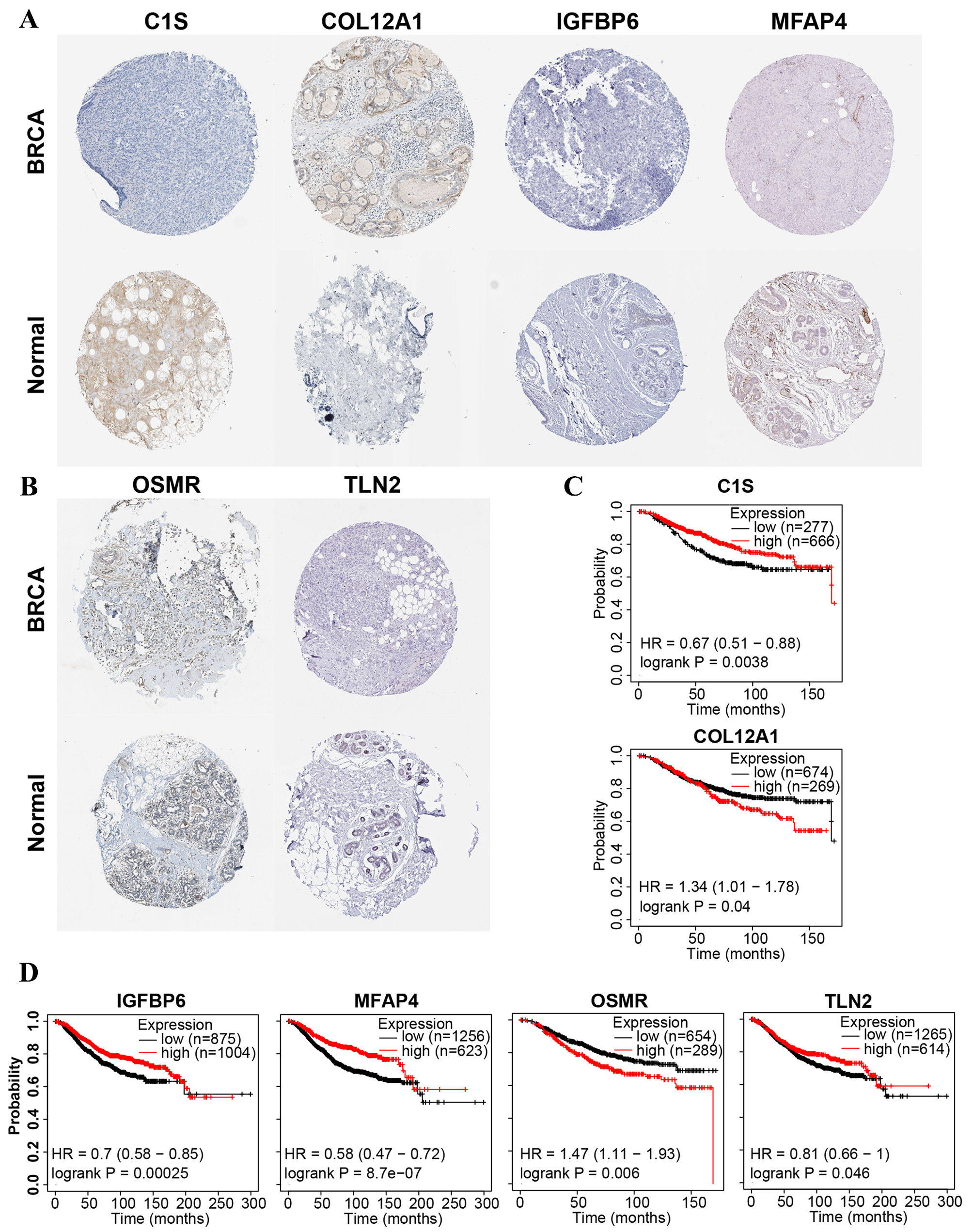

Supplement: Supplementary Figure 1 — WGCNA was performed to cluster samples and assess the correlation between modular genes and CAF infiltration. (A, C) Samples were clustered and those with branch positions above the red line were removed. (B, D) Modular genes were strongly positively correlated with CAF infiltration. The results displayed on the left panel (A, B) are based on the MCPcounter algorithm, and those displayed on the right panel (C, D) are based on the xCELL algorithm. [file DataSheet_1.zip › Supplementary Figure S9.tif]
